# Aptamers for Anti-Viral Therapeutics and Diagnostics

**DOI:** 10.3390/ijms22084168

**Published:** 2021-04-17

**Authors:** Tae-Hyeong Kim, Seong-Wook Lee

**Affiliations:** 1 Department of Molecular Biology, Dankook University, Cheonan 31116, Korea; kth6086@naver.com; 2 Department of Life Convergence, Research Institute of Advanced Omics, Dankook University, Yongin 16890, Korea; 3 R&D Center, Rznomics Inc., Seongnam 13486, Korea

**Keywords:** virus, aptamer, diagnostics, therapeutics, SELEX, influenza, HIV, HCV, COVID-19

## Abstract

Viral infections cause a host of fatal diseases and seriously affect every form of life from bacteria to humans. Although most viral infections can receive appropriate treatment thereby limiting damage to life and livelihood with modern medicine and early diagnosis, new types of viral infections are continuously emerging that need to be properly and timely treated. As time is the most important factor in the progress of many deadly viral diseases, early detection becomes of paramount importance for effective treatment. Aptamers are small oligonucleotide molecules made by the systematic evolution of ligands by exponential enrichment (SELEX). Aptamers are characterized by being able to specifically bind to a target, much like antibodies. However, unlike antibodies, aptamers are easily synthesized, modified, and are able to target a wider range of substances, including proteins and carbohydrates. With these advantages in mind, many studies on aptamer-based viral diagnosis and treatments are currently in progress. The use of aptamers for viral diagnosis requires a system that recognizes the binding of viral molecules to aptamers in samples of blood, serum, plasma, or in virus-infected cells. From a therapeutic perspective, aptamers target viral particles or host cell receptors to prevent the interaction between the virus and host cells or target intracellular viral proteins to interrupt the life cycle of the virus within infected cells. In this paper, we review recent attempts to use aptamers for the diagnosis and treatment of various viral infections.

## 1. Introduction

A virus is an organism at the boundary between living and non-living. Viruses are infectious pathogens and proliferate by infecting living organisms. Simple in form, viruses consist of nothing but some genetic material and an encompassing protein shell. Viruses have no means of self-reproduction, and therefore must invade host cells in order to use the host’s molecular machinery to reproduce, after which the newly created viruses go on to infect other host cells. Proliferation in this way can cause either acute disease or chronic disease depending on how long the virus leaves the host cell intact. Various diseases such as coronavirus 19 (COVID19), acquired immune deficiency syndrome (AIDS), and hepatitis are caused by viruses. Viral diseases are often associated with various symptoms such as fever, cough, and weakness, but some present with few or no symptoms, making identification and treatment harshly difficult. Therefore, methods to either improve diagnostic accuracy or provide viable treatment options are of intense interest within the healthcare community.

Viruses sometimes cause fatal damage to the human body and without proper treatment, can lead to death. Therefore, many drugs and vaccines have been developed for the treatment of viral diseases. When a host comes in contact with a viral particle, the processes of attachment, uncoating, genome replication, assembly, and release quickly follow. Currently, most drugs target this process at some level to suppress the life cycle of the virus, thereby limiting host damage and ideally restricting the spread of infection. In addition, some treatments attempt to treat the virus by inducing the host’s own immune response, however, as viruses tend to mutate quickly, many easily avoid the effects of highly targeted drugs and the host’s immune system. In cases such as these, it becomes difficult to treat the viral infection without causing toxicity to host cells.

There have been many attempts to minimize side effects and accurately select specific targets for the treatment of different viral infections. In this review, we address aptamers as a potential alternative for both the treatment and diagnoses of viral infections. Aptamers are small single-stranded RNA or DNA molecules that can be rapidly selected through SELEX (Systematic Evolution of Ligands by EXponential enrichment). Aptamers can be identified against various targets with high affinity and specificity and are easy to synthesize and modify. Due to these clear advantages, many attempts have been made to develop aptamers as antiviral drugs and diagnostic substances.

## 2. Aptamers

Aptamers are small single-stranded oligonucleotides first developed by Szostak and Ellington through an in vitro selection of RNA molecules that bound to various organic dyes [1]. Aptamers form a three-dimensional shape and specifically bind to target molecules [2]. They are also referred to as chemical antibodies because their function is so similar to that of normal antibodies, but there are important differences. While antibodies recognize and bind to protein epitope sequences, aptamers recognize and bind based on the 3D structure of the target molecule [3]. Aptamers are produced through chemical synthesis, and thus batch reproducibility and stability are high, manufacturing is inexpensive, and immunogenicity is typically minimal [4]. The following table is a comparison between aptamers and antibodies (Table 1).

**Figure 1:**
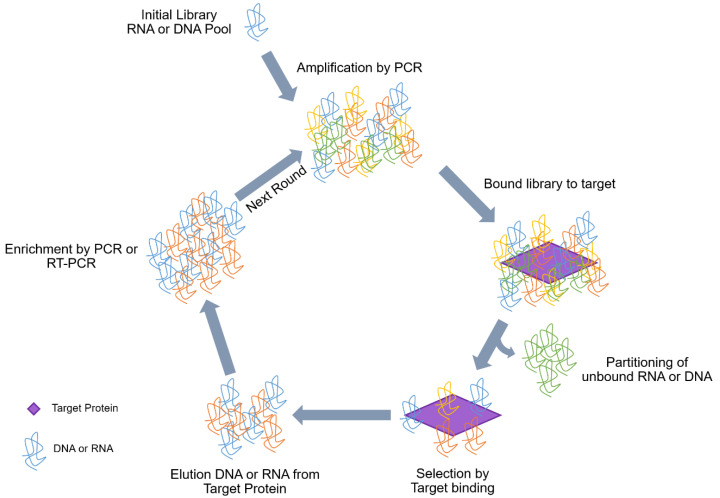
Scheme of the general systematic evolution of ligands by exponential enrichment (SELEX) procedure. The SELEX procedure is a repeated process as follows. (1) incubate library with target; (2) remove unbound oligonucleotides; (3) Elute target binding oligonucleotides; (4) enrichment by polymerase chain reaction (PCR) or reverse transcription-polymerase chain reaction (RT-PCR).

Aptamers are developed by Systematic Evolution of Ligands by EXponential enrichment (SELEX), a method developed by Gold and Tuerk [5]. The first step of SELEX involves the synthesis of a fully or partially randomized oligonucleotide sequence library of some length flanked by defined regions which allow PCR amplification of those randomized regions [1,5] (Figure 1). This oligonucleotide library contains 10^14^–10^15^ nucleic acid variants of 30–100 random nucleotides flanked by constant sequences at both ends. The randomized oligonucleotides have unique sequences that acquire a three-dimensional structure depending on the experimental conditions (pH, ionic strength, temperature, etc.), or the presence of specific ligands [6]. These characteristics mean that aptamers can bind to targets with both high affinity and specificity. The second step of SELEX involves incubation of the random library with the target molecule (protein, cell, virus, etc.); this step is performed in optimized conditions (temperature, pH, salt concentration, etc.) [6]. During this step, a subpopulation of the library interacts with the target molecule and an enriched subpopulation is then isolated. These isolated sequences (target bound sequences) are eluted and amplified for the next cycle while the unbound sequences are removed. This cycle is repeated 8 to 15 times until the best target specific sequence pool is obtained.

## 3. Aptamers for Double-Stranded DNA Viruses

### 3.1. Aptamers for Human Papillomavirus (HPV)

Human papillomavirus (HPV) is a non-enveloped DNA virus of the family Papilomaviridae. Most HPV infections show no symptoms and resolve within two years, however, in some cases, infection can generate warts or precancerous lesions and these sites increase the risk of cancer in the cervix, vulva, vagina, penis, and anus [7]. HPV is the main cause of cervical cancer and two strains (HPV-16, HPV-18) account for 70% of HPV-related cervical cancers. During the viral life cycle, two viral proteins (E6, E7) act as oncoproteins. These two proteins bind to p53 and RB, respectively, to promote their degradation and activate the cell cycle of the host cells. Three vaccines (Gardasil, Gardasil 9, and Cervarix) are available to prevent infection of HPV, and they have been shown to effectively block initial infection from diverse HPV types including HPV16 and HPV18. However, outside of those vaccines, there are no adequate therapeutics to cure HPV infection or the resulting cancers.

#### 3.1.1. Aptamers for HPV Diagnostics

Toscano-Garibay et al. isolated an RNA aptamer against the HPV-16 E7 oncoprotein. The isolated RNA aptamer is specifically bound to E7 in a clamp-like manner and might be useful for the detection of HPV infection [8]. Leija-Montoya et al. developed RNA aptamers against HPV-16 L1 virus-like particles (VLPs). They suggested that the isolated aptamers showed specific and stable binding to HPV-16 VLPs even in biofluid protein mixes, suggesting they could be used as a diagnostic tool [9]. For more effectively detecting HPV, several other aptamer-based systems have been developed. Trausch et al. isolated a slow off-rate modified DNA aptamer (SOMAmer) against HPV-16 VLPs. When this aptamer was used in ELISA, the aptamer showed very high specificity. They demonstrated that this assay format offered more time and required fewer resources than traditional ELISA [10]. Aspermair et al. developed a reduced graphene oxide-based field-effect transistor (rGO-FET) for detecting the HPV E7 protein in saliva using RNA aptamers. They showed that the aptamer-functionalized rGO-FET could detect aptamer-HPV-16 E7 protein binding in real-time and in human samples with a detection limit of 100 pg/mL [11].

#### 3.1.2. Aptamers for HPV Therapeutics

Aiming to create treatments specific for HPV infection, several groups have developed various aptamer-based therapeutics. Nicol et al. isolated RNA aptamers against the HPV-16 E7 oncoprotein. They showed that the isolated aptamers could inhibit the interaction between E7 and RB [12] and the RNA aptamer induced apoptosis combined with the loss of E7 when transfected into HPV-16-transformed cells [13]. The same group investigated the effects of the RNA aptamers on E7 localization. They found that, without the aptamer, E7 localized to the plasma membrane. They then showed that the aptamer enhanced E7 localization in the ER and that the reduction of E7 was not related to proteasomal degradation [14]. Gourronc et al. isolated RNA aptamers against HPV-16 E6/E7 transformed tonsillar epithelial cells (HTECs) by using cell SELEX. The isolated aptamers effectively internalized into HPV-16 E6/E7-HTECs, suggesting that they could be used for delivering therapeutic agents [15]. Belyaeva et al. developed RNA aptamers against the PDZ-binding motif of the HPV-16 E6 oncoprotein. When these aptamers were transfected into the HPV16 transformed cells, they induced apoptosis through inhibition of the interaction between E6 and PDZ1, not between E6 and p53 [16]. Valencia-Resendiz et al. showed that RNA aptamers specific for HPV16 L1 virus-like particles (VLPs) inhibited the HPV16 infection step when 293TT cells were infected by HPV16 pseudovirus [17].

### 3.2. Aptamers for Herpes Simplex Virus (HSV)

Herpes simplex viruses 1 and 2 (HSV-1 and HSV-2) are linear double-stranded DNA-enveloped viruses that belong to the Herpesviridae family. They can infect epithelial tissues and invade the nervous system where they enter a latent stage of infection. Both viruses persist in the human body by hiding in the cell bodies of neurons and when activated move from neurons to skin where virus replication and shedding occurs [18]. Most HSV infections do not require treatment, and antiviral therapy is typically only performed when the lesions persist for a long time and are accompanied by other symptoms and complications. However, no treatments specific to HSV have been developed to date.

In attempts to develop antiviral agents against HSV, several groups have isolated aptamers against the HSV envelope glycoprotein (gD). Moore et al. developed DNA aptamers against HSV-2 gD. These aptamers could bind to gD and block HSV2 infection by blocking the interaction of gD and the various entry receptors (Nectin1 and HSV1 target cell receptors (HVEM)) [19]. Gopinath et al. isolated RNA aptamers that were specifically bound to the HSV-1 gD protein. These aptamers specifically bound to HSV-1 gD and had no effect on the HSV-2 gD. The authors truncated the aptamer to a 44-mer which could bind to HSV-1 gD with high affinity and inhibit the gD–HVEM interaction. Furthermore, this aptamer had anti-HSV-1 activity [20]. Yadavalli et al. also isolated a DNA aptamer against the HSV-1 gD protein. Their 45-mer DNA aptamer showed specific binding to HSV-1 gD with high affinity and its administration showed significant decreases in viral entry and replication in vitro, ex vivo, and in vivo [21].

## 4. Aptamers for Positive Sense Single-Stranded RNA Viruses

### 4.1. Aptamers for the Hepatitis C Virus (HCV)

HCV is a small, enveloped virus of the family Flaviviridae. HCV is the cause of hepatitis C and can also cause liver fibrosis, cirrhosis, and liver cancer, making HCV infection a major global public health concern. HCV has positive-sense single-stranded RNA as a genome that contains about 9.6 kilobases which has an untranslated (UTR) region (at 5′ and 3′ ends) and a coding region for a viral polyprotein. This polyprotein is composed of four structural proteins (C, E1, E2 and p7), and six non-structural proteins (NS2, NS3, NS4A, NS4B, NS5A and NS5B). The first stage of HCV infection is viral attachment to the target cells through their glycoproteins and the cell-surface molecules CD81, LDL receptor, SR-BI, DC-SIGN, Claudin-1, and Occludin [22]. After attachment, the virus enters the target cells through clathrin-mediated endocytosis at which point the virus sheds its coating and releases its genome. The released viral RNA is directly translated into polyprotein precursors that are cleaved into single proteins by host and viral proteases. The generated proteins play a role in the replication of the viral genome and the assembly and maturation of viral particles. Finally, the virion is released from cells by exocytosis or transferred to other cells through a cell-free mechanism. The high variability of HCV helps it to evade the host’s immune response and leads to a poor prognosis even with anti-HCV treatment. Accordingly, HCV has various genotypes, and thus accurate diagnosis and treatment are vital.

#### 4.1.1. Aptamers for HCV Diagnostics

Aptamers to be used as HCV diagnostics tools have been developed for HCV detection in both the early stages and in immunosuppressed patients. Lee et al. developed a chip-based detection system for HCV using RNA aptamers that are specifically bound to the HCV core antigen. They immobilized 2′-F RNA aptamers in a 96-well plate and detected the HCV core antigen from the patient’s sera by Cy3-labeled secondary antibodies [23]. Chen et al. developed an HCV detection system based on a sandwich-ELISA method using DNA aptamers that are specifically bound to HCV envelope glycoprotein E2. They observed a good correlation in HCV patients between the HCV E2 antigen-aptamer assay and assays for HCV RNA quantities or HCV antibody detection [24]. Park et al. reported a new aptamer-based assay system suitable for measuring the infectious titer of HCV using DNA aptamers against HCV E2. They developed a system they named the Enzyme-Linked Apto-Sorbent Assay (ELASA) and the readout value of HCV ELASA linearly correlated with the infectious dose of the HCV samples [25]. Wang et al. developed a rapid, easy-to-use, and chip-based diagnostic platform using lateral flow strips composed of thiol-DNA aptamers against the HCV core antigen. They showed positive coincidence rates with ELISA and strip detection when compared with the HCV RNA amplification assay [26]. Ghanbari et al. developed an ultra-sensitive aptasensor based on a graphene quantum dot (GQD) nanocomposite for the detection of the HCV core antigen. They showed rapid, selective, and sensitive detection of the HCV core antigen in human serum through electrochemical impedance spectroscopy (EIS) and the detection limit was 3.3 pg/mL [27]. Pleshakova et al. developed an HCV core antigen detection system using an atomic force microscope chip (AFM-chip) with immobilized DNA aptamers. They showed the aptamers were successfully utilized as probe molecules for HCV core antigen detection in human serum at concentrations from 10^−10^ M to 10^−13^ M [28,29].

#### 4.1.2. Aptamers for HCV Therapeutics

There are currently no effective vaccines or treatments for HCV. Generally, HCV treatments combine protease inhibitors, like Telaprevir (TVR) and Boceprevir (BOC), with pegylated-interferon (PEG-IFN) and Ribavirin. Fortunately, newly developed treatments using pan-genotypic direct-acting antivirals (DAAs) are short duration (12~24 weeks) with few side effects and have about a 90% of cure rate, regardless of HCV genotype [30]. Unfortunately, these treatments are often too expensive, frequently cause side effects, and are susceptible to new resistant viruses. Therefore, the development of more effective new DAA treatments is still necessary. Many aptamers have been developed for the treatment of HCV to date [31].

##### Therapeutic Aptamers for Targeting the HCV Viral Genome

The 5′ and 3′ UTR of the HCV genome have highly conserved sequences and structured regions which are strongly related to the translation and replication of HCV. The 5′ UTR contains the Internal Ribosome Entry Site (IRES) domain which plays an important role in translation initiation by facilitating the ribosome’s recognition of the HCV viral genome. Additionally, the 3′ UTR has a region called the cis-acting replication element (CRE) which is also essential for viral replication.

Kikuchi et al. selected an RNA aptamer that is specifically bound to the HCV IRES. The selected aptamer bound to the target sequences via RNA–RNA interactions and the aptamers that targeted domain IIId showed strong inhibition of translation [32,33]. In addition, aptamers that targeted domain II showed a 20~40% inhibition rate in translation inhibition through loop–loop interactions [34,35]. The authors conjugated the two aptamers and showed increased inhibition of IRES-dependent translation [36].

Fukuda et al. isolated RNA aptamers specific for the 3′ X tail of the HCV RNA which had the potential to block HCV replication [37]. Marton et al. developed RNA aptamers that specifically bound to 3′ UTR CRE and CRE-5BSL3.2 domain. The aptamers selected for binding to the 3′ UTR CRE inhibited the HCV RNA’s replication up to 80% [38]. RNA aptamers specific for the CRE-5BSL3.2 domain interfered with the binding of the NS5B protein to CRE and induced a significant reduction in HCV replication [39].

Another target is the minus-IRES ((-) IRES) at 3′ end of the negative strand of the HCV RNA. This structure is essential for HCV replication because the viral RNA polymerase NS5B recognizes this site as the initiation site for the synthesis of the positive-strand RNA. Konno et al. isolated RNA aptamers specific for HCV (−) IRES domain I. They showed that this aptamer inhibited up to 50% of the NS5B-mediated positive-strand RNA synthesis [40,41,42].

##### Therapeutic Aptamers for Targeting HCV Viral Proteins

Aptamers for targeting HCV Structural Proteins (E1, E2, and core)

The HCV structural proteins E1 and E2 are good targets for HCV therapeutics because of their essential role in target recognition during HCV entry. Chen et al. developed DNA aptamers specific for HCV glycoprotein E2 that showed a high affinity for genotypes 1a, 1b, and 2a, and strongly inhibited HCV infection [24]. Yang et al. developed DNA aptamers specific for the HCV E1 and E2 proteins and showed that the developed aptamers inhibited HCV infection in an infectious cell culture system [43]. The HCV core protein is a viral nucleocapsid protein that is essential for viral assembly. Shi et al. developed aptamers against this HCV core protein and showed that while the developed aptamers had no effect on HCV RNA replication, they did inhibit the production of infectious viral particles in vitro cell culture system [44].

Aptamers for targeting HCV Nonstructural Protein 2 (NS2)

The HCV NS2 protein is a viral autoprotease that plays a key role in the life cycle of HCV by mediating the cleavage between NS2 and NS3, making NS2 an important target for HCV treatments. Gao et al. developed DNA aptamers specific for the HCV NS2 protein. They showed that selected aptamers had an anti-viral effect through binding to the N-terminus of NS2 that blocked the interaction of NS2 with NS5A [45].

Aptamers for targeting HCV Nonstructural protein 3 (NS3)

The HCV NS3 is a multifunctional protein that has three enzymatic activities. First, NS3 has serine protease activity which is encoded at its N-terminal region. In addition, NS3 has nucleoside triphosphatase (NTPase) and helicase activities at its C-terminal region. Therefore, the HCV NS3 protein is an essential protein for the viral life cycle and an important target for HCV treatments. Kumar et al. developed RNA aptamers specific for HCV NS3 protein and showed that selected aptamers inhibited both the proteolytic and helicase activity of HCV NS3 [46]. Hwang et al. also developed RNA aptamers against the NS3 helicase domain. They showed that RNA aptamers partially inhibited RNA synthesis in vitro and in HCV subgenomic replicon cells [47]. Nishikawa and colleagues have developed diverse RNA aptamers that specifically bind to the HCV NS3 protein. To inhibit the protease activity of NS3, they developed RNA aptamers that are specifically bound to the NS3 protease domain which have potential as anti-NS3 molecules [48,49]. In addition, they conjugated aptamers with cis-acting genomic human hepatitis delta virus (HDV) ribozymes and showed inhibition of protease activity of the HDV ribozyme-aptamer in HeLa cells [50,51]. The same group also developed RNA aptamers against the HCV NS3 helicase domain. The selected aptamers showed strong inhibitory effects in vitro, and the aptamer’s stem-loop part was required for helicase inhibition [52,53]. They also designed bi-functional RNA aptamers for obtaining dual inhibition effects of both the protease and helicase activities. They showed inhibition of NS3 protease activity in living cells and HCV replication in vitro by conjugating NS3 protease aptamers and helicase aptamers [54,55,56].

Aptamers for targeting HCV Nonstructural Protein 5A (NS5A)

HCV NS5A is a dimeric zinc-binding metalloprotein capable of binding viral RNA, Core, and host factors like lipid droplets, and is essential for HCV production and replication. Yu et al. developed HCV NS5A-specific DNA aptamers and showed that selected aptamers inhibited HCV RNA replication and infectious virus production by blocking the interaction of NS5A with the Core protein. Importantly, the aptamers did not induce interferon beta (IFN-β) or interferon-stimulated genes (ISGs) [57].

Aptamers for targeting HCV Nonstructural Protein 5B (NS5B)

HCV NS5B is an RNA-dependent RNA polymerase protein (RdRp) which is essential for the synthesis of positive-sense HCV genomic RNA and negative-sense RNA templates. Because of NS5B’s role in HCV replication, NS5B is the main target for HCV treatments using aptamers. Biroccio et al. developed an RNA aptamer against a truncated HCV NS5B∆55 that did not have a C-terminal region. They showed that the HCV NS5B polymerase was efficiently inhibited by the selected aptamer. Interestingly, the mechanism of inhibition turned out to be noncompetitive with template RNA, suggesting that the aptamer and template RNA did not bind to the same site [58]. Bellecave et al. developed DNA aptamers against HCV NS5B. The selected aptamers competed with RNA templates’ 3′-ends of positive and negative sense HCV RNA for binding to the polymerase and inhibited the initiation steps of HCV RNA replication [59,60]. Jones et al. developed DNA aptamers specific for HCV genotype 3a NS5B. These aptamers were the first genotype-specific aptamers and showed NS5B inhibitory effects in genotypes 1a, 1b and 3a [61]. Kanamori et al. developed GC-rich RNA aptamers specific for HCV NS5B. These aptamers contained the CGGG motif that exists in the stem structure of the NS5B coding RNA (5BSL3.2). They showed that these aptamers impaired binding and inhibited the activity of NS5B [62]. Lee et al. developed RNA aptamers against HCV NS5B where they selected aptamers with 2′ hydroxyl (OH) nucleotides or modified fluoropyrimidines (F) to avoid degradation. Both kinds of aptamers inhibited the replication of HCV RNA in cell culture without cellular toxicity [63]. They also conjugated the truncated 2′ F-RNA aptamers with cholesterol to facilitate delivery in cell culture and in vivo to great effect. Aptamers that were conjugated with cholesterol (chol-aptamer) efficiently entered the cell and inhibited HCV RNA replication, without any induction of innate immune response-related genes and caused no abnormalities in mice when administered systemically. Moreover, they evaluated the pharmacokinetics of the chol-aptamer in vivo wherein they demonstrated that the cholesterol conjugation showed dose proportionality, extended the time that the aptamer was present in plasma, and increased aptamer exposure throughout the body. Of Note, the intravenous administration route increased the period of time the chol-aptamer was present in plasma compared to intraperitoneal injection, suggesting intravenous injection was the most appropriate treatment route in vivo [64].

### 4.2. Aptamers for Zika Virus (ZIKV)

The Zika virus (ZIKV) is a member of the virus family Flaviviridae. ZIKV is an enveloped virus with a genome of single-stranded positive-sense RNA about 10 kilobases in size. This genome is directly translated into a viral polyprotein that encodes three structural proteins and seven non-structural proteins. Zika’s replication depends on the synthesis of double-stranded RNA from its single-stranded positive-sense RNA genome, after which the replication of the new single-stranded positive-sense RNA proceeds [65]. ZIKV causes a disease called Zika fever, which typically has no or only mild symptoms, however, when ZIKV infects pregnant women, it also infects the developing fetus, potentially causing microcephaly, severe brain malformations, and other birth defects. Additionally, in rare cases, adult infection can lead to Guillain–Barré syndrome [66]. There are no effective vaccines or treatments for ZIKV to date, though several groups have developed ZIKV diagnostic systems and therapeutics using aptamers.

Lee et al. developed an aptamer-based ELISA assay specific for ZIKV using anti-ZIKV non-structural protein 1 (NS1) DNA aptamers. ZIKV NS1 is essential for RNA replication and immune evasion [67]. In the report, their system showed a detection limit of 100 ng/mL of NS1 [68]. Kim et al. developed an aptamer-based immunoassay to detect Zika virus in serum and urine. They used a DNA aptamer specific for ZIKV NS1 along with peptides that are specifically bound to ZIKV. They showed that the in silico modeled aptamer discriminated ZIKV from Dengue virus (DENV) and the detection limit was 1 × 10^4^ tissue culture infective dose (TCID) 50/mL [69]. Dolai et al. developed paper-based sensor potentiometry using ZIKV specific DNA aptamers. They showed that this system could detect the whole ZIKV with a minimum sensitivity of 0.26 nV/Zika and a minimum detectable signal (MDS) of 2.4 × 10^7^ ZIKV [70].

### 4.3. Aptamers for Dengue Virus (DENV)

Dengue virus (DENV) is a single-stranded positive-sense RNA enveloped virus also of the family Flaviviridae. Based on the type of envelope proteins, DENV has four genotypes called DENV-1, DENV-2, DENV-3, and DENV-4. DENV envelope proteins bind to cellular receptors which allows the virus to enter the cell and release its viral RNA. Like other Flaviviridae viruses, DENV viral RNA is also translated into a polyprotein (include three structural and seven non-structural proteins) and is processed by cellular or viral proteases. After that, processes such as replication, maturation, and release take place. While DENV is the known cause of dengue fever, many DENV infections are understood to be asymptomatic or subclinical [71]. Interestingly, severe forms of dengue do exist, and this is especially true for persons who had been previously infected with DENV and get infected again later with another serotype. Previously generated antibodies to the old DENV serotype interfere with the immune response to the newly infected DENV serotype, which leads to more viral entry and uptake [72]. Several aptamers have been developed for the detection and treatment of DENV.

#### 4.3.1. Aptamers for DENV Diagnostics

For DENV diagnostics, Fletcher et al. developed an aptamer-based biosensor using DNA aptamers for the DENV genome. They showed that the biosensor was able to detect sequences derived from each of the four DENV serotypes with high specificity [73]. For the same purpose, Basso et al. developed an immunosensor using hybrid nanomaterials. This immunosensor was composed of γ-Fe_2_O_3_ (SAMN) nanoparticles and modified with gold nanoparticles (AuNPs) conjugated to aptamers [74]. This method efficiently detected all four DENV serotypes.

#### 4.3.2. Aptamers for DENV Therapeutics

To target DENV viral proteins, Gandham et al. developed DNA thioaptamers that specifically bound DENV-2 envelope protein domain III (ED3). They showed that the aptamers could bind to adjacent neutralizing antibody binding sites and expected that the aptamers inhibited infection by blocking DENV entry into the host cells [75]. Chen et al. also developed DNA aptamers targeting DENV-2 ED3 which bound to a highly conserved loop between the βA and βB strands of ED3. Although the aptamer was developed to target DENV-2 specifically, they found it could neutralize all four serotypes of DENV effectively [76]. Jung et al. developed an RNA aptamer targeting the methyltransferase (MTase) of DENV-2 and DENV-3. They showed that the selected 45-mer truncated RNA aptamer could specifically bind to DENV MTase and competitively inhibit MTase methylation activity [77]. Another target for aptamer development is the DENV viral genome. Cnossen et al. developed DNA aptamers targeting the 5′-UTR of DENV and demonstrated that the stem-loops of the aptamers could interact with the viral genome. These are the first RNA aptamers targeting functional RNA elements of the DENV genome and may have potential as future therapeutic and diagnostic tools [78].

### 4.4. Aptamers for Japanese Encephalitis Virus (JEV)

The Japanese encephalitis virus (JEV) is a single-stranded positive-sense RNA enveloped virus of the family Flaviviridae. Based on the type of envelope proteins, JEV has five genotypes. The JEV viral genome, much like others in the same family, encodes several structural and non-structural proteins and the JEV life cycle is similar to other Flaviviridae viruses. JEV infects the central nervous system and causes Japanese encephalitis (JE). About 67,900 cases of JEV-related JE are reported annually and its mortality ranges from 20 to 30% [79]. Currently, there are no effective therapeutics to treat JEV, though some aptamers have been considered. Han et al. developed an RNA aptamer against the JEV methyltransferase (MTase), which is essential for viral replication and catalyzes the methylation of the viral RNA cap. They identified several specific RNA aptamers modified with 2′-O-methyl pyrimidines against JEV MTase. Specifically, they found a truncated 24-mer RNA aptamer efficiently inhibited JEV replication and production in cell culture [80].

### 4.5. Aptamers for Tick-Borne Encephalitis Virus (TBEV)

The tick-borne Encephalitis Virus (TBEV) is a positive-sense single-stranded RNA virus also of the family Flaviviridae. Its 11 kilobase genome contains a 5′-cap, 5′-UTR, 3′-UTR, and a single open reading frame (ORF). The ORF encodes three structural proteins and seven non-structural proteins and the life cycle of this virus is again similar to other Flaviviridae viruses. TBEV is the cause of tick-borne encephalitis (TBE) and this disease typically targets the central nervous system (CNS) [81]. There are no specific antiviral therapeutics for TBEV because the specific immunoglobulin used in clinical practice has disadvantages such as limitation in its production by the limited number of available donors and risk of side effects like anaphylactic shock. Kondratov et al. obtained DNA aptamers against the surface envelope proteins of TBEV. They showed that treatment with these aptamers better suppressed TBEV infection than the current commercial human immunoglobulin against TBEV (NPO Microgen, Russia) [82].

### 4.6. Aptamers for Norovirus (NoV)

Norovirus (NoV) is a non-enveloped virus of the family Caliciviridae and has a single-stranded positive-sense RNA as a genome. Its RNA genome encodes a viral polyprotein that is processed into six non-structural proteins and one structural protein. It has at least seven genogroups (GI, GII, GIII, GIV, GV, GVI, and GVII) including various genotypes within each group, and among them, GI and GIV can infect humans [83]. NoV is a cause of gastroenteritis and annually infects about 685 million people, 200,000 of whom die each year worldwide, especially in developing countries [84]. Despite significant concern about the virus from the public, conventional diagnosis systems are severely limited. NoV infection recovers on its own without special treatment in those who do recover, but for those who will not, there are no specific antiviral agents for NoV.

Escudero-Abarca et al. developed DNA aptamers against human NoV. Isolated aptamers bound to NoV GII.2 and GII.4 strains with high affinity and the virus was found in contaminated lettuce through an aptamer magnetic capture (AMC) method with RT-qPCR. They demonstrated that the isolated aptamers were potentially useful for NoV capture and detection in a variety of sample types [85]. The same group also isolated DNA aptamers against the capsid P domain of human NoV strain GII.4. By using the same method as above, they showed that the isolated aptamers had a strong binding reactivity to GI.7, GII.2, two GII.4 strains, and GII.7 virus-like particles (VLPs) [86]. Liu et al. also isolated aptamers against the P domain of human NoV GII.4 strain by using an in-situ target-capture approach. They selected aptamers from the 30,622,226 tested sequences and could detect NoV in clinical samples [87].

To create a system for diagnosing NoV, Giamberadino et al. developed an aptasensor using DNA aptamers. They developed aptamers against murine NoV, but found it could cross-react with human NoV strain GII.3. Aptamers were immobilized on the electrochemical sensor using a gold nanoparticle-modified screen-printed carbon electrode (GNPs-SPCE) and could detect NoV with a detection limit of approximately 180 viral particles [88]. Chand et al. developed a microfluidic platform integrated with a graphene-gold nano-composite aptasensor for the detection of NoV. They used a viral capsid-specific aptamer and obtained a detection range between 100 pM and 3.5 nM [89]. Kim et al. developed an aptasensor for monitoring NoV. They used a modified DNA aptamer linked to five guanines that were specific for the NoV GII capsid protein. The modified DNA aptamer enhanced the sensitivity of the biosensor based on intra chemiluminescent resonance transfer (Intra-CRET). The biosensor accurately detected the NoV GII capsid in tap water without any additional time-consuming and tedious procedures [90].

### 4.7. Aptamers for Coronavirus (CoV)

Coronavirus (CoV) is a positive-sense single-stranded RNA virus that belongs to the family Coronaviridae with a genome of about 30 kilobases in size. CoV’s genome is organized as 5′-leader-UTR-replicase (ORF1ab)—spike (S)—envelope (E)—membrane (M)—nucleocapsid (N)—3′UTR-poly (A)—tail, and thus can act like a messenger RNA (mRNA) [91]. The virus releases its genome into host cells where it can be directly translated by the host cell’s ribosomes and makes two large overlapping polyproteins. The viral polyproteins are processed to each viral protein that has an essential role in the viral life cycle. CoV infects the respiratory tract and the results of that infection are diverse, ranging from mild to lethal. CoV is drawing particular attention currently as it is the cause of the largest pandemic of the 21st century.

In 2003, an outbreak of severe acute respiratory syndrome (SARS) begun in Asia and quickly spread. The virus named SARS-CoV infected more than 8000 people, about 10% of whom died [92]. In September 2012, a new type of CoV was identified that was named Middle East respiratory syndrome coronavirus (MERS-CoV). Up to December 2019, 2468 people were infected with MERS-CoV, and its mortality rate was 34.5%. In December 2019, pneumonia was reported in Wuhan, China, which was found to be due to CoV infection. The virus was designated SARS-CoV2 and is the cause of Coronavirus disease 2019 (COVID-19). The virus was identified to have about 70% genetic similarity to SARS-CoV [93]. By 11 April 2021, there were more than one hundred million people infected with many millions dead. Vaccines based on various platforms including mRNA, DNA, recombinant proteins, or viruses, etc., are currently being actively developed, however, since variant strains of SARS-CoV2 are frequently generated, any vaccine that expects to perform well must have efficacy against a wide range of genotypes. Despite the fact that this pandemic is still ongoing, there continue to be no efficient treatments that work widely for COVID-19.

#### 4.7.1. Aptamers for SARS-CoV

Jang et al. developed RNA aptamers against the SARS-CoV NTPase/Helicase (nsP10). They showed that the isolated aptamers efficiently inhibited the double-stranded DNA unwinding activity of the helicase by up to 85%, but showed only a slight effect on ATPase activity [94]. Shum et al. developed DNA aptamers against the SARS-CoV Helicase. The isolated aptamers contained diverse secondary structures with G-quadruplexes or non-G-quadruplexes and they showed only non-G-quadruplex aptamers caused specific inhibition of helicase activities [95]. For detecting SARS-CoV, Ahn et al. developed an RNA aptamer-based SARS-CoV detection system using an RNA aptamer against the SARS-CoV nucleocapsid (N) protein. They used a chemiluminescence immunosorbent assay and a nanoarray aptamer chip and could detect the N protein at concentrations as low as 2 pg/mL [96]. Cho et al. isolated DNA aptamers also against the SARS-CoV N protein. They demonstrated that these aptamers efficiently facilitated the detection of the SARS-CoV N protein when compared with an N protein antibody, suggesting the aptamers could represent a good alternative probe for SARS-CoV detection [97]. Roh et al. isolated RNA aptamers against the SARS-CoV N protein and developed a SARS-CoV detection system using quantum dot (QD)-conjugated RNA aptamers on a chip. Using this system, they showed that the SARS-CoV N protein was detectable at concentrations as low as 0.1 pg/mL [98].

#### 4.7.2. Aptamers for SARS-CoV2

With the ongoing pandemic in mind, Song et al. developed DNA aptamers against the SARS-CoV2 herpes simplex virus receptor-binding domain (RBD) of the spike glycoprotein (S protein). They isolated two aptamers that may have partially identical binding sites at the human SARS-CoV2 receptor angiotensin-converting enzyme 2 (ACE2) on the SARS-CoV2 RBD, and predicted the structure of the aptamer–RBD binding complex. The structure showed that aptamer could bind to RBD by hydrogen bonding. They also demonstrated that these aptamers were useful for the diagnosis and treatment of SARS-CoV2 [99] Zhang et al. developed DNA aptamers against the SARS-CoV2 nucleocapsid (N) protein. They found that four pairs of aptamers could bind to the N protein through a sandwich-type interaction. Using the aptamers, they created an ELISA assay and colloidal gold immunochromatographic strips with the ability to detect the N protein at levels of tens of pM [100].

## 5. Aptamers for Negative Sense Single-Stranded RNA Viruses

### 5.1. Aptamers for Influenza Virus

The influenza virus is a member of the family Orthomyxoviridae. Those in this family are enveloped viruses with a segmented negative single-stranded RNA as the genome. There are four types of this virus, type A, type B, type C, and type D, however, only types A and B are clinically relevant to humans [101]. The influenza virus has two membrane glycoprotein components, hemagglutinin (HA) and neuraminidase (NA), associated with its envelope. This virus has eight single-stranded RNA fragments as its genome where each fragment has one or two genes, and the eight RNA fragments encode a total of 15 viral proteins. Among these eight proteins, HA and NA are highly variable surface proteins. HA helps the virus invade host cells and NA helps the virus release from the host cells. In total, 18 variants of HA and 11 of NA have been identified, and the host organism and infectivity of the influenza virus are determined by the combination of these two proteins. Only the subtypes H1, H2, H3, N1, and N2 have been found in humans to date.

The high infectivity and variability of the influenza virus have caused serious pandemics to occur several times throughout the 20th and 21st centuries. In 1918, the Spanish influenza virus killed 20–50 million people. In 1957, the Asian influenza virus recorded approximately 1.1 million deaths. Additionally, in 1968, the Hong Kong influenza virus caused about 1 million people to die worldwide [102,103]. Finally, in 2009, a new influenza A virus subtype H1N1 (pH1N1) emerged and with its high infectivity, it rapidly spread worldwide and caused the first pandemic in the 21st century [104,105]. Therefore, in view of the high infectivity and variability of the influenza virus, methods to rapidly diagnose and treat it are critical components for controlling the spread and preventing future pandemics.

#### 5.1.1. Aptamers for Influenza Virus Diagnostics

Rapid diagnosis of influenza and accurate classification of a subtype are vital due to its high infectivity and mutagenicity. While antibodies are typically used for the detection of these viruses, most current antibodies differentiate only between influenza types A and B. In cases like this, the advantages of aptamers become quickly apparent (target specificity, broad target molecule, etc.) and offer a potential alternative method of identification. Anti-influenza aptamers mainly target viral particles or hemagglutinin (HA), and more than 40 aptamers have been developed to date. Especially in recent years, due to the rapid spread of the H1N1 virus and the high fatality rate of the H5N1 subtype of the avian influenza virus (AIV), the influenza virus has become the main target for the development of aptamers. Aptamers targeting H1, H3, H5, H9, or Ha (type B) for HA and an aptamer targeting the H5N1 viral particle have been developed just in recent years [106].

##### Aptamers for Influenza A Virus Diagnostics

Because of its high infectivity and mutagenicity, most of the aptamers developed thus far are anti-influenza A aptamers. Cui et al. developed a diagnostic system using Quantum dots (QD) [107]. They combined QD with an influenza type A specific DNA aptamer and confirmed that the virus was labeled through fluorescence imaging (FI) and transmission electron microscopy (TEM). Zhang et al. developed a fluorescence polarization platform using an influenza A/H1N1 virus-specific aptamer [108]. Tseng et al. developed an automatic micro fluidic system for fluorescence-based detection of influenza A/H1N1 using a sandwich-based anti-H1N1 virus DNA aptamer [109] where the entire processing time was about 30 min. This method was considerably faster than conventional viral culture methods and the detection limit was also improved with 10^3^ times the sensitivity of conventional serological diagnosis. Wang et al. also developed a microfluidic device using DNA aptamers [3] that took advantage of the property that a single universal aptamer causes conformational changes at different ion concentrations, allowing for the simultaneous identification of three different virus types (H1N1, H3N2, and type B) in just 20 min. Lee et al. developed an aptamer-based electrochemical sensor using an anti-H1N1 virus aptamer that could distinguish the H1 subtype from the H5 subtype as a molecular probe [110]. To do this, they immobilized an aptamer on a Si-wafer, ran an enzyme-linked immunosorbent assay (ELISA), and finally performed field emission scanning electron microscopy (FE-SEM). Moreover, they also immobilized the aptamer on an indium thin oxide-coated surface and performed cyclic voltammetry (CV). Through this process, they could detect target protein molecules up to the nanomolar scale. Bai et al. developed a multivalent binding-based electrical impedance detection system using an anti-H1N1 virus DNA aptamer [111]. They identified a DNA aptamer through SELEX and used this aptamer in a sandwich enzyme-linked oligonucleotide assay (ELONA) and electrochemical impedance (EIS) aptasensor. With these, they could detect a target at levels as low as 0.3 ng/μL via ELONA and 0.9 pg/μL via EIS aptasensor. Bhardwaj et al. developed a label-free electrochemical biosensor using a DNA aptamer specific for the H1N1 virus HA stem region [112]. They used an indium thin oxide electrochemical sensor for the detection system and performed CV and EIS measurements which allowed them to detect the target virus at levels of 3.7 pfu/mL. Chen et al. developed a Surface-enhanced Raman scattering (SERS)-based aptasensor using an anti-H1N1 DNA aptamer [113]. To do this, they fabricated a three-dimensional (3D) nano-popcorn plasmonic substrate using the surface energy difference between a perfluorodecanethiol (PFDT) spacer and the Au layer. Quantitative evaluation of the H1N1 virus was achieved using the decrease in Raman peak intensity resulting from the release of Cy3-labeled aptamer DNAs from the nano-popcorn substrate surfaces via the interaction between the aptamer DNA and H1N1 virus. By using 3D nano-popcorn, this system had the advantage of being ultrasensitive and reproducible.

Le et al. developed an aptamer-based biosensor using a specific RNA aptamer for the H3N2 virus [114] which combined the aptamer with a gold nanoparticle. The resulting particle could bind the target virus and formed a gold nano shell on the virus envelope which allowed for simple visual detection and was very low cost. Chen et al. developed a magnetic microparticle-based colorimetric platform using a specific DNA aptamer for the H3N2 virus [115]. In a glucose solution, the DNA aptamer-linked magnetic beads (MB) captured the target molecule and formed gold nanoparticles, inducing a color change in the solution thereby facilitating detection. Le et al. developed a dual recognition element lateral flow assay (DRELFA) for detecting the H3N2 virus [116]. They used both an antibody and an aptamer to compensate for the shortcomings of each molecule, which specifically bind to virus hemagglutinin. They combined a biotin-labeled aptamer and a gold nanoparticle-labeled antibody and when the virus attached to the aptamer and antibody, it formed a complex and then could be captured by streptavidin. This method was about 50 times more sensitive than using RNA or antibodies only. Kukushkin et al. developed a SERS aptasensor using specific a DNA aptamer for the H3N2 virus [117]. They achieved a high sensitivity by using a sandwich of primary aptamers to influenza virus and secondary aptamers. The primary aptamers were first attached to the metal particles of the SERS substrate, and influenza viruses were captured and bound with secondary aptamers labelled with Raman-active molecules. The detection limit was as low as 10^−4^ hemagglutinin units (HAU)/probe.

##### Aptamers for Avian Influenza Virus (AIV) Diagnostics

The H5N1 virus, a subtype of AIV, also requires a fast and accurate diagnosis because it is potentially fatal and spreads quickly within human populations. Therefore, many diagnostic systems have been developed using various aptamers. Bai et al. developed a portable Surface Plasmon Resonance (SPR) biosensor for the rapid detection of AIV H5N1 using a specific DNA aptamer [118]. This biosensor was fabricated using selected aptamers and the immobilized aptamers captured the AIV H5N1 virus in a sample solution. Wang et al. developed a Hydrogel based quartz crystal microbalance (QCM) aptasensor using a specific DNA aptamer for the H5N1 virus [119]. This aptasensor was based on an ssDNA crosslinked polymeric hydrogel. Unfortunately, QCM-based aptasensors are not suitable for in-field use because the QCM is highly sensitive to environmental noise. Lum et al. developed an impedance aptasensor with microfluidic chips using a specific DNA aptamer for the H5N1 virus [120]. The gold surface of an interdigitated microelectrode was modified using streptavidin and a biotinylated aptamer was then immobilized on the electrode surface using biotin–streptavidin binding. This aptasensor required 30 min per assay and had a detection limit of 0.0128 HAU. Pang et al. developed a fluorescent aptasensor system based on core–shell nanoparticle metal-enhanced fluorescence (MEF) for H5N1 virus detection [121]. Anti-HA aptamers were immobilized on the surface of the Ag@SiO2 nanoparticles which then acted as the metal-enhanced fluorescence (MEF) sensing platform. The entire detection process could be completed within 30 min. Karash et al. developed an impedance aptasensor with gold nanoparticles using a specific aptamer for the H5N1 virus [122]. In this system, streptavidin was immobilized on a microelectrode surface and a biotin-labeled H5N1 aptamer was bound to the immobilized streptavidin. The bound aptamer then captured the target and the detection time of aptasensor without amplification was less than one hour with a detection limit of 0.25 HAU for the pure virus.

Nguyen et al. developed a specific sandwich-type SPR-based biosensor for the detection of H5Nx whole viruses [123]. They use a pair of aptamers selected against a mixture of H5Nx whole viruses using Multi-GO SELEX. This dual aptamer-based system’s sensitivity was increased by more than 50-fold compared to a single-aptamer system. Kwon et al. developed an aptamer-functionalized field-effect transistor (FET) as a label-free sensor for AIV H5N1 detection using a specific aptamer for HA [124]. This aptamer was immobilized on a gold microelectrode that was connected to the gate of a reusable FET transducer and when the target protein bound to the aptamer, a signal response was generated. The signal of the aptamer-based FET biosensor increased linearly with HA protein concentration in a range of 10 pM to 10 nM with a detection limit of 5.9 pM.

#### 5.1.2. Aptamers for Influenza Virus Therapeutics

Anti-viral aptamer therapeutics can target viral proteins or related host cellular factors. Most influenza therapeutics being currently developed also target these molecules, and most developed aptamers mainly target hemagglutinin (HA) [125]. HA plays a key role in viral entry as it allows recognition of the target cell’s sialic acid-containing receptors. Once bound to a receptor, it facilitates the entry of the viral genome into the target cells. Thus, most of the aptamers targeting HA block viral entry into host cells. Jeon et al. developed an influenza A virus HA-specific DNA aptamer [126]. Gopinath et al. developed an RNA aptamer specific for the influenza B virus’ HA, influenza A H3N2s’ HA, and influenza A H1N1 [127,128,129]. Cheng et al. developed an avian influenza virus (AIV) H5N1 HA-specific DNA aptamer [130]. Choi et al. and Park et al. developed an AIV H9N2 HA-specific DNA aptamer and an AIV H5 HA-specific RNA aptamer, respectively [131,132]. Musafia et al., Kwon et al., and Suenaga et al. developed an influenza virus HA-specific DNA aptamer, AIV H5 HA-specific RNA aptamer, and an AIV H5N1 HA-specific RNA aptamer, respectively [133,134,135]. Zhang et al. developed an AIV H9N2 HA-specific DNA aptamer [136]. Li et al. developed an influenza virus A H1N1 HA-specific DNA aptamer [137]. All these aptamers targeted the influenza virus surface protein, HA, meaning these aptamers bound to HA thereby blocking HA activity, resulting in a disruption to viral entry into the host cells.

Another aptamer target is the NS1 protein, a viral nonstructural protein encoded by the NS gene segments of the influenza virus. NS1 prevents the polyadenylation of cellular mRNAs in order to escape the antiviral responses of the host cells. Moreover, the RNA binding domain of NS1 is able to target RIG-I, which is responsible for the cellular interferon (IFN) response. Finally, NS1 inhibits the host’s innate immune response by suppressing the induction of IFNs. Therefore, blocking NS1 activity could be a potential strategy for antiviral agents against the influenza virus. Woo et al. developed a specific DNA aptamer for the influenza A virus NS1 protein [138]. In their report, by blocking the NS1 protein with the specific aptamer, they induced IFN-β thereby facilitating the host’s innate immune response. They also found that aptamer was able to inhibit viral replication without affecting cell viability.

Another target for the development of influenza virus aptamers is the PA endonuclease. PA plays an essential role in viral RNA transcription and replication by forming the heterotrimeric polymerase complex together with the PB1 and PB2 subunits. PA cleaves host mRNAs downstream of their mRNA cap structures, which are then recognized and bound by PB2 [139]. The N-terminal domain of the PA subunit (PAN) is highly conserved among different subtypes of influenza virus, which strongly supports PA as an attractive target for anti-influenza aptamers. Yuan et al. developed DNA aptamers targeting the intact PA protein or the PAN of the H5N1 virus [140]. They developed nine aptamers and among them, four PAN-specific aptamers were found that inhibited both endonuclease activity and H5N1 virus infection. In addition, one exhibited cross-protection against infection by H1N1, H5N1, H7N7, and H7N9 influenza viruses, with a 50% inhibitory concentration (IC50) around 10 nM. Moreover, they predicted the structure of aptamer–PA binding complex. The structure showed that aptamer could bind to the enzyme pocket of PA endonuclease.

Another strategy is to target influenza virus-related host cellular factors. The mRNAs of the influenza virus possess a 5′ cap structure and a 3′ poly (A) tail that makes them structurally indistinguishable from cellular mRNAs. However, selective translation of viral mRNAs occurs in infected cells through a discriminatory mechanism, whereby the viral polymerase and NS1 interact with components of the translation–initiation complex, such as the eIF4GI and PABP1 proteins [141,142,143]. Therefore, inhibition of the viral protein–translation factor interactions could be another strategy for anti-influenza therapeutics. Rodriguez et al. developed DNA aptamers specific for the translation initiation complex component PABP1 [144]. Two aptamers were developed and both of them inhibited the interaction of the viral polymerase with the eIF4GI translation–initiation factor and also inhibited the association of NS1 with PABP1 and eIF4GI.

### 5.2. Aptamers for Rift Valley Fever Virus (RVFV)

The rift valley fever virus (RVFV) belongs to the family of Phenuiviridae and is an enveloped negative single-stranded RNA virus. RVFV has three segmented RNAs as a genome and they encode six major viral proteins. This virus has an outer lipid envelope with two glycoproteins (G(N) and G(C)) that are required for cell entry. RVFV infection is the cause of several severe diseases such as hemorrhagic fever syndrome, meningoencephalitis, hepatitis, and can severely affect the eyes [145]. Currently, there are no licensed anti-RVFV vaccines or therapeutics. Ellenbecker et al. developed RNA aptamers against the nucleocapsid protein (N) of RVFV. The N protein of RVFV is an RNA binding protein that is essential for the production of new viral particles as it protects the viral genome from degradation during replication [146]. They screened the structure of isolated RNA sequences in silico and selected RNA aptamers that were similar to N-binding RNAs. When these aptamers were administered to cells prior to infection by RVFV, they effectively inhibited viral replication [147].

### 5.3. Aptamers for Severe Fever with Thrombocytopenia Syndrome Virus (SFTSV)

The severe Fever with Thrombocytopenia Syndrome Virus (SFTSV), also called Dabie bandavirus, is a tick-borne virus in the family Phenuiviridae [148]. It has three segmented negative-sense RNAs as a viral genome (large (L), medium (M) and small (s)) which encode several viral proteins, though only five of those proteins have been identified to date. SFTSV is the main cause of severe fever with thrombocytopenia syndrome (SFTS) and this disease has a fatality rate of 12–30%. Its clinical symptoms are fever, vomiting, diarrhea, multiple organ failure, thrombocytopenia, leukopenia, and elevated liver enzyme levels. These symptoms are most severe in patients who have multiple organ failure and a high SFTSV concentration in their blood [149]. Considering the seriousness of the symptoms caused by this virus, the development of appropriate diagnosis and treatment options is in high demand. Yeom et al. developed a diagnostic application using DNA aptamers against the nucleocapsid protein (N) of SFTSV. The N protein of SFTSV is the most immunodominant protein with few mutations [150]. In their system, they conjugated DNA aptamers and proteins with liposomes and encapsulated them with horseradish peroxidase (HRP) for catalytic signal amplification. This system’s detection limit was 0.009 ng/mL, and N of SFTSV was successfully detected in diluted human serum [151].

### 5.4. Aptamers for Ebola Virus (EBOV)

The Ebola virus (EBOV) is an RNA virus of the family Filoviridae and belongs to the genus Ebolavirus. It has a negative-sense single-stranded RNA as a genome and the viral genome contains seven genes arranged as follows: 3′-UTR-NP-VP35-VP40-GP-VP30-VP24-L-5′-UTR [152]. Five viruses in this genus are known to cause Ebola virus disease (EVD) or Ebola hemorrhagic fever (EHF). EVD has a high risk of death at about 25–90% with an average of 50% due to its severe symptoms like low blood pressure from extreme fluid loss. Moreover, some of these strains were the cause of the EBOV epidemic in west Africa in 2013–1015. A vaccine specific to EBOV was approved in the United States in 2019, but there are no specific therapeutics for EBOV infection.

Binning et al. developed RNA aptamers against EBOV viral protein 35 (eVP35). eVP35 is a multifunctional double-strand RNA binding protein that is essential for viral replication, innate immune evasion, and pathogenesis. eVP35 has a dsRNA binding interferon (IFN) inhibitory domain (IID) at its C-terminal for eVP35-mediated IFN antagonism and viral polymerase co-factor functions. The authors showed that the isolated aptamer could bind to eVP35 IID and disrupt the eVP35–nucleoprotein (NP) interaction. In addition to its ability to antagonize the eVP35–NP interaction, the isolated aptamer also inhibited the EBOV polymerase complex [153]. Tanaka et al. developed DNA aptamers against EBOV viral protein 24 (eVP24). eVP24 is important for the inhibition of IFN and promotes viral infection by accelerating viral budding and assembly. Additionally, eVP24 can bind to karyopherin alpha 1 (KPNA) and inhibit IFN signaling. The authors showed that the isolated aptamers could bind to eVP24 at sub-nanomolar levels and effectively inhibit it from binding to KPNA1 [154].

Shubham et al. developed a 2′fluoro pyrimidine (2′FY)-modified RNA aptamer against the soluble glycoprotein (sGP) of EBOV. sGP is a good biomarker for EBOV because it is secreted in abundance into the bloodstream, even during the early stages of infection. The isolated aptamer was specifically bound to sGP with high affinity and was demonstrated to be an excellent diagnostic tool for the detection of EBOV infection [155]. Hong et al. developed DNA aptamers against EBOV GP and NP using a highly efficient aptamer selection platform in which they used magnetism-controlled selection chips to select aptamers. Selected aptamers effectively bound to EBOV GP and NP and were successfully applied to the detection of EBOV [156].

## 6. Aptamers for Single-Stranded RNA Viruses with DNA Intermediate

### 6.1. Aptamers for Human Immunodeficiency Virus (HIV)

HIV is a lentivirus (a subgroup of retroviruses) that causes acquired immunodeficiency syndrome (AIDS) [157]. It is an enveloped virus composed of two copies of positive-sense single-stranded RNA that encodes the virus’s nine genes. HIV infects human immune cells such as CD4+ T cells, macrophages, and dendritic cells. When CD4+ T cell numbers decrease to a critical level, cell-mediated immunity is lost, and the body becomes more susceptible to opportunistic infections. The HIV virion enters macrophages and CD4+ T cells via endocytosis. The first step of viral entry involves attachment of the CD4 binding domains of viral gp120 to CD4. Once gp120 is bound to the CD4 protein, the envelope complex undergoes a structural change that allows gp120 to interact with the target chemokine receptor, which finally allows for cell membrane penetration via the N-terminal fusion peptide gp41 [158]. After endocytosis, the HIV RNA and enzymes (reverse transcriptase, integrase, ribonuclease, and protease) are injected into the cell. Once the viral components are injected into the host cell, an enzyme called reverse transcriptase releases the positive-sense single-stranded RNA genome from the attached viral proteins and copies it into a complementary DNA (cDNA) molecule [159]. The process of reverse transcription is extremely error-prone and as a result, numerous mutations occur in the produced HIV genomes which allows the virus to effectively evade the host’s immune system. After replication of the viral genome, the cDNA and its complement form a double-stranded viral DNA (provirus) that is integrated into the target cell’s chromosome inside the nucleus. During viral replication, the integrated DNA provirus is transcribed into RNA, and then undergoes RNA splicing to produce mature mRNAs. These mRNAs are exported from the nucleus into the cytoplasm where they are then translated into the regulatory proteins Tat and Rev. The newly produced Rev proteins move to the nucleus, and then bind to full-length, unspliced copies of viral RNA which then allows them to leave the nucleus [160]. These full-length RNAs function as new copies of the viral genome or are then translated to produce the structural proteins Gag and Env. Gag and Env proteins bind to copies of the viral RNA genome, and package them into new viral particles. The provirus’s integration into the host genome can lead to either chronic or acute infection. Chronic infection results in viral latency, and the integrated provirus can remain hidden for many years during which it can replicate and form new viral particles at any moment. In acute infection, the HIV genome is actively transcribed and translated. As a result, new viral particles are made and the host cells are destroyed. The newly made viral particles are then free to infect new cells. Considering the high variability and lethality of HIV, accurate and rapid diagnosis and appropriate treatment are of paramount importance.

#### 6.1.1. Aptamers for HIV Diagnostics

Using aptamers may also be a good option to diagnose HIV. Various attempts to use aptamers for the diagnosis of HIV have been reported. For instance, Pavski et al. developed an affinity capillary electrophoresis/laser-induced fluorescence (CE/LIF) assay using a specific DNA aptamer for HIV-1’s reverse transcriptase (RT) as a probe [161]. An aptamer–HIV-1 RT complex was readily formed, and the calibration curves were linear up to 50 nM (6 µg/mL) HIV-1 RT. Mascini’s lab developed two different kinds of aptamer-based biosensors using a specific RNA aptamer for the HIV-1 Tat protein [162,163]. The first biosensor was optimized using piezoelectric quartz-crystals as transducers and the aptamer was immobilized on the gold electrode of the crystal. For the other biosensor, the aptamer was immobilized on the gold surface of surface plasmon resonance (SPR) chips to develop an SPR-based biosensor. Both systems used a biotin–streptavidin interaction for detecting the virus. Ruslinda et al. developed a diamond field-effect transistor (FET)-based RNA aptamer sensing system for detecting the HIV-1 Tat protein [164]. Stable immobilization was achieved via RNA aptamers and obtained high sensitivity for the HIV-1 Tat protein. Unfortunately, this biosensor was complex and its use cost-prohibitive. Babamiri et al. developed a molecularly imprinted polymer (MIP) electrochemiluminescence (MIP-ECL) sensor for highly sensitive and selective HIV-1 gene detection using Europium sulfide nanocrystals (EsNCs) as the signal producing compound [165]. For this, they used the HIV aptamer as a template. This ECL biosensor was applied to the detection of HIV DNA in real human serum samples and satisfactory results were obtained with both high sensitivity and selectivity. Caglayan et al. developed a spectrophotometric ellipsometry-based Tat-protein RNA-aptasensor using a specific RNA aptamer for the HIV-1 Tat protein [166]. They showed high HIV-Tat protein detection with both the spectroscopic ellipsometry (SE) and with the surface plasmon resonance-enhanced total internal reflection ellipsometry (SPReTIRE).

#### 6.1.2. Aptamers for HIV Therapeutics

There is no complete cure for HIV/AIDS, however, current treatments do manage to slow the progression of the disease and reduce the risk of death. These treatments are called antiretroviral therapies and are created through the use of a combination of several antiretroviral drugs (cART). cART is usually a combination of three or more medications from several different drug classes. Unfortunately, cART has numerous shortcomings such as severe side effects and high cost. Thus, aptamers have been considered as potential alternative agents in cART to overcome these limitations. Many aptamers have been developed specifically targeting HIV genomes, HIV proteins, or cellular factors related to HIV infection.

##### Therapeutic Aptamers for Targeting the HIV Viral Genome

The HIV genome consists of two identical single-stranded RNAs. Each RNA has a long terminal repeat (LTR) region at its 5′ and 3′ end. The 5′ LTR region acts as a promotor for the transcription of viral genes. If the functions of HIV LTR are inhibited by disrupting their structure or by blocking their interaction with viral or cellular factors, it may affect HIV’s life cycle. Srisawat et al. developed RNA aptamers that could bind to the LTRs of HIV-1 DNA [167]. The aptamer’s conserved segments formed duplexes via Watson-Crick base-pairing with the preferred sequences in one strand of the DNA, assuming the aptamer invaded the duplex. The aptamers could then inhibit the transcription process of the HIV genome. Sanchez-Luque et al. developed specific RNA aptamers against the 5′ LTR region of the HIV-1 genome [168]. These aptamers inhibited more than 75% of HIV-1 production in a human cell line. In addition, the short form of these aptamers showed inhibition of HIV-1 production close to 85%.

##### Therapeutic Aptamers for Targeting HIV Viral Proteins

Aptamers for targeting HIV Reverse Transcriptase (RT)

The RT of HIV plays an important role in viral genome replication. RT has DNA polymerase activity and also plays a role as an RNase H. RT can copy the genome to either a DNA or an RNA template and can cleave RNA only when the RNA is part of an RNA/DNA duplex. Therefore, these two enzymatic activities convert the RNA into a linear double-stranded DNA [169]. Many aptamers have been developed for inhibiting these activities to date.

Chaloin et al. developed a pseudoknot RNA aptamer specific for the HIV-1 RT [170]. Transient intracellular expression of the aptamer showed inhibition of HIV particle release of >75% and the subsequent virus production of human T-lymphoid C8166 cells was reduced by >75%. Held et al. also developed pseudoknot RNA aptamers specific for HIV-1 RT [171,172]. In their study, they showed polymerase-independent RNase H activity was the most resistant to long-term aptamer suppression, and RNA-dependent DNA polymerization was the most susceptible. Joshi et al. also developed RNA aptamers specific for the HIV-1 RT. When those aptamers were expressed in target cells, the aptamers more efficiently inhibited HIV replication than short hairpin RNAs (shRNAs), especially in higher multiplicities of infection (m.o.i) [173,174]. Kissel et al. developed a DNA aptamer specific for HIV-1 RT and showed that the aptamer inhibited recombinant RT cloned from diverse branches of the primate lentiviral family (HIV-1, HIV-2, and SIVcpz). In addition, they predicted aptamer–RT binding complex. The structure showed that aptamer could bind to RT by forming a double helix [175,176]. DeStefano et al. developed a DNA aptamer directed against the reverse transcriptase of HIV HXB2. They showed the aptamer competed with the natural template for the binding site in the enzyme which inhibited viral replication [177]. Michalowski et al. identified three DNA aptamers that contained a bimodular structure comprised of a 5′-stem-loop module connected to a 3′-G-quadruplex. In their report, they demonstrated that these aptamers inhibited the RT from diverse primate lentiviruses with low nM IC50 values [178]. Lange et al. developed an HIV-1 RT specific RNA aptamer modified with a hammerhead ribozyme which had a self-cleavage effect [179]. Clonal stable cell lines expressing aptamers from these modified constructs strongly suppressed infectious virus production. Shiang et al. developed anti-HIV-1 RT aptamers conjugated with gold nanoparticles [180]. In their report, the nuclease-stable G-quadruplex structure of the aptamer-gold nanoparticles showed efficiency in inhibiting viral replication with a significantly decreased infectivity (40.2%). Whatley et al. developed RNA aptamers that bound the RT of HIV-1 and identified aptamer transcripts with non-pseudoknot motifs with conserved unpaired UCAA elements [181]. The aptamers inhibited RT in primer extension assays with IC50 values in the low nmol/l range and inhibited viral replication more than previously studied aptamers. Lange et al. found that HIV inhibition by non-pseudoknot RNA aptamers against RT required encapsidation and exhibited broad-spectrum HIV inhibition, suggesting these aptamers had the potential to overcome aptamer-specific viral resistance [182]. Nguyen et al. identified aptamer structural elements critical for HIV inhibition and established the role of the signature UCAA bulge motif in the RT–aptamer interaction [183]. In this study, they predicted the structure of aptamer–RT binding complex. The structure showed that the UCAA bulge might not be buried in the complex but rather remains exposed and shapes the aptamer’s 3D structure. In addition, an aptamer enhanced the proteolytic cleavage of precursor p66/p66 by the HIV-1 protease, suggesting an acceleration of RT maturation and hence interference with HIV replication by the aptamer.

For specifically targeting RNase H activity, Andreola et al. developed two DNA aptamers [184]. The selected aptamers had a G-rich sequence and could form G-quartets. These aptamers inhibited the RNase H activity of HIV-1 RT and had no effect on cellular RNase H. Solutrait et al. also developed a DNA aptamer for HIV-1 RNase H inhibition [185]. Their aptamer also had G-rich sequences able to form G-quartets and could inhibit HIV-1 replication.

Aptamers for targeting HIV Integrase (IN)

Integrase (IN), which catalyzes the integration of newly synthesized double-stranded viral DNA into the host’s genome, is also a good target for anti-HIV aptamer therapeutics [186]. Métifiot et al. developed DNA aptamers targeting HIV-1 RT’s RNase H, but surprisingly, while the RNase H activity was unaffected by these aptamers, the HIV-1 integrase was strongly inhibited [187]. Ojwnang et al. identified a DNA aptamer (T30177) targeting the HIV-1 genome, and this aptamer also inhibited HIV-1 integrase activity [188]. This aptamer was the first IN inhibitor tested in clinical trials (Zintevir™, developed by Aronex Pharmaceuticals, The Woodlands, TX, USA) [189]. Faure-Perraud et al. developed a guanine-quadruplex aptamer that inhibited HIV-1 integrase activity. This aptamer severely affected the proviral integration step independently of the effect on viral entry [190]. Virgilio et al. improved the activity of the anti-HIV-1 integrase aptamer through the replacement of aptamer’s individual thymidines with 5-hydroxymethyl-2′-deoxyuridine residues (H) [191]. In this study, the modified aptamer showed a higher ability to inhibit the HIV IN than the unmodified aptamer. Moreover, they also predicted the structure of aptamer–IN binding complex. The structure showed that the residues H in the modified aptamers are able to establish several contacts with both chains A and C of the target HIV-1 IN.

Aptamers for targeting HIV Protease (PR)

HIV protease (PR) has a dual purpose; the precursor HIV PR is responsible for catalyzing its own production into mature PR through PR auto-processing, where the mature PR then hydrolyzes peptide bonds on the Gag-Pol polyproteins at nine specific sites and produces functional proteins (RT, IN, and etc.) [192]. Few PR targeting aptamers have been designed to date, though Duclair et al. did develop RNA aptamers against HIV-1 PR [193]. These aptamers inhibited both in vitro PR activity and viral replication in cells.

Aptamers for targeting HIV Nucleocapsid (NC) protein

The nucleocapsid (NC) protein of HIV-1 plays an important role in the encapsidation of viral RNAs and assembly of viral particles [194]. The NC protein is highly conserved, and thus may be a good target for anti-viral therapeutics. Kim et al. developed an RNA aptamer that bound to the mature form of the NC protein [195]. They suggested that the RNA aptamer acted as an inhibitor of viral packaging. Kim et al. also demonstrated that when this aptamer was expressed in cells, it inhibited the packaging of viral genomic RNA [196].

Aptamers for targeting HIV Surface Envelope Glycoprotein (gp120)

Gp120 is essential for the HIV virus to enter target cells as it plays a vital role in HIV’s attachment to specific cell surface receptors. Interestingly, many neutralizing antibodies bind to variable regions of gp120, which is likely the reason this protein has such a high mutation rate [197]. For blocking HIV entry, several small aptamers have been developed. James’s lab developed 2′F-RNA aptamers targeting the HIV-1 gp120 [198,199]. These not only bound gp120 with high affinity but also neutralized HIV-1 infectivity in human peripheral blood mononuclear cells (PBMCs) by more than 1000-fold. These aptamers could also bind to gp120’s CCR5 binding site which further neutralized viral infection [200,201]. Khati et al. assessed the activity of a shortened synthetic derivative of the B40 aptamer, called UCLA1 [202]. UCLA1 is tightly bound to a consensus HIV-1 subtype C gp120 and neutralized isolates of the same subtype with 50% inhibitory concentrations (IC50) in the nanomolar range.

Aptamers for targeting the HIV Gag protein

Another target viral protein is the HIV Gag polyprotein. Gag polyproteins are processed by protease into matrix proteins, capsid proteins, spacer peptides, and nucleocapsid proteins. Two aptamers have been developed targeting the Gag protein. Lochrie et al. developed RNA aptamers against the HIV-1 gag protein [203], which bound the region of the matrix (MA) protein or nucleocapsid (NC) protein. Ramalingam et al. recently identified a new RNA aptamer against the HIV-1 Gag protein [204] which showed a 20-fold inhibition in the extracellular capsid levels and significantly reduced cellular levels of Gag mRNA.

Aptamers for targeting the HIV Rev protein

The HIV Rev protein mediates nuclear export of unspliced and partially spliced viral RNAs. Rev assembles on a 351-nt Rev response element (RRE) within viral transcripts and induces the host’s nuclear export mechanism [205]. Aptamers can act as a Rev-binding element (RBE) and therefore anti-Rev aptamers are likely a good strategy for anti-HIV therapeutics. Symensma et al. developed RNA aptamers that were specific for HIV-1 Rev [206]. These aptamers were found to be functionally equivalent to the wild-type element and demonstrated that cellular factors did not directly influence the Rev–RBE interaction. Konopka et al. also developed a Rev-binding aptamer to inhibit HIV replication [207]. This aptamer suppressed HIV production from HIV proviral clones. Finally, Dearborn et al. identified small RNA aptamers that competed with the RRE for Rev binding, and predicted the structure of aptamer–Rev binding complex [205]. The structure showed that Rev’s ARM interacts with the major groove of the aptamer. These aptamers were structurally similar to the RRE high-affinity site and were effective in vitro by blocking Rev self-assembly. Thus, they propose that aptamers can inhibit HIV-1 replication by interfering with Rev–RRE, Rev–Rev, and possibly Rev–host protein interactions.

##### Therapeutic Aptamers for Targeting HIV Related Cellular Factors

Another strategy for blocking HIV infection is to target the host’s cellular factors which to date, has mainly meant targeting host cell receptors. HIV uses C–C chemokine receptor type 5 (CCR5) or C–X–C chemokine receptor type 4 (CXCR-4) as co-receptors to invade target cells. Of note, mutations in CCR5 confer immunity to HIV in humans. Therefore, CCR5 is an essential factor for HIV’s entry and likely a good target for the treatment of HIV. Zhou et al. developed RNA aptamers against CCR5 [208]. They showed that the aptamers efficiently neutralized the HIV-1 infectivity of R5 strains with an IC50 of about 170~350 nM.

Another target is nucleolin (NCL). NCL is a multifunctional nucleolar protein. In cellular fractions, NCL complexes with CD4, CXCR4, and CCR5, thus, NCL is most heavily involved in the early stage of HIV’s entry. If there are no cellular receptors like CD4 and CXCR4/CCR5 on the cell surface, HIV-1 attachment occurs through interactions with heparan sulfate proteoglycans and cell-surface-expressed NCL. Perrone et al. developed a G-rich aptamer against NCL that formed a stable G-quadruplex structure and showed that this aptamer efficiently inhibited HIV-1 attachment/entry into host cells [209].

Another potential target is human cyclin T1 (CycT1). The regulatory cyclin, CycT1, is a host factor essential for HIV-1 replication in CD4 T cells and macrophages. CycT1 complexes with Positive Transcription Elongation Factor b (P-TEFb) to facilitate HIV replication [210]. Um et al. developed an RNA aptamer with specific affinity toward CycT1 and showed that the aptamer inhibited binding of cyclin-dependent kinase 9 (Cdk9) to CycT1, resulting in the inhibition of HIV transcription [211].

## 7. Aptamers for Single-Stranded DNA Viruses with RNA Intermediate

### 7.1. Aptamers for Hepatitis B Virus (HBV)

Hepatitis B virus (HBV) is a partially double-stranded DNA virus that belongs to the Hepadnaviridae family and is classified into eight genotypes from A to H. The HBV viral DNA genome is rendered fully double-stranded DNA by cellular DNA polymerases and transcribed by cellular RNA polymerases. The transcribed viral RNAs are translated into the viral proteins essential for the virus’s life cycle. HBV is the cause of hepatitis B, which can be either acute or become chronic leading to liver cirrhosis and hepatocellular carcinoma. Despite an effective vaccine that prevents HBV infection, it is still a global health concern due to the lack of adequate treatments.

#### 7.1.1. Aptamers for HBV Diagnostics

For effectively detecting HBV, Suh et al. developed a detection system for the HBV surface antigen (HBsAg) using specific RNA aptamers. HBsAg is the main element of the HBV particle and thus the main target of diagnostic tests. To create their system, they combined the conventional competitive binding assay with fluorescence resonance energy transfer (FRET) and aptamers. They demonstrated that their method was approximately 40-fold better than that of the most widely used conventional detection assays [212]. Xi et al. developed HBsAg-specific DNA aptamers attached to carboxylated magnetic nanoparticles (MNPs). With these, they created a chemiluminescence aptasensor for detecting HBsAg by using the selected aptamers. This system’s detection limit was five-fold better than the conventional enzyme-linked immunosorbent assay (ELISA) used in hospitals [213]. Huang et al. developed fluorescence-based aptasensors for detecting HBV e antigen (HBeAg) using specific DNA aptamers. They showed that a fluorescent-labeled HBeAg aptamer could serve as the molecular recognition element and efficiently detected HBeAg in blood serum within 2 min [214].

#### 7.1.2. Aptamers for HBV Therapeutics

For developing effective aptamer therapeutics, Feng et al. generated RNA aptamers against the HBV polymerase protein (P protein). During viral replication, the interaction between the P protein and the RNA stem-loop on the pregenomic (pg) RNA is essential. They showed that the isolated aptamers inhibited viral pgRNA packaging and DNA synthesis by blocking P protein binding to pgRNA [215]. Zhang et al. developed DNA aptamers against the HBV core protein that is essential for the assembly of the nucleocapsid of HBV. They showed that the isolated aptamers efficiently inhibited the assembly of the nucleocapsid and reduced extracellular HBV DNA [216]. Orabi et al. developed DNA aptamers against the matrix binding domain (MBD) on the HBV capsid. They showed that the selected aptamers inhibited HBV virion production up to 47% and this result suggested the importance of MBD in virion assembly [217]. Zheng et al. showed that an HBsAg-specific aptamer bound to agarose through a DNA tetrahedral structure could remove HBsAg as an immunosorbent. These highly structured molecules effectively removed HBsAg in cells without any cytotoxicity and thus have potential as an HBV treatment [218].

## 8. Conclusions

In the past 20 years, many studies have been conducted that attempted to treat or diagnose specific diseases using aptamers. In particular, the advantages of aptamers have enabled many studies on viral diseases that would have been otherwise difficult to conduct using older methods (Table 2). Overall, aptamers may represent a plausible alternative method for the treatment and diagnosis of many viral diseases for which no specific therapeutics or diagnostics yet exist.

In order to effectively treat viral diseases, a quick and accurate diagnosis in the early stages of infection is essential. There have been many attempts to use aptamers for virus detection which represent potentially simpler, faster, and cheaper systems than those otherwise available. These aptamers systems require more intensive development and larger-scale trials to ensure their effectiveness and safety, and improvements over time are rapidly converging on these goals. Take the detection systems using field-effect transistors as an example, these systems started as complex and expensive systems using diamond [164]. However, later, simpler and more cost-effective aptamer-based systems using new materials such as graphene were developed. The newly developed systems had advantages like faster response times, increased ease of use, and the potential for miniaturization.

**Figure 2:**
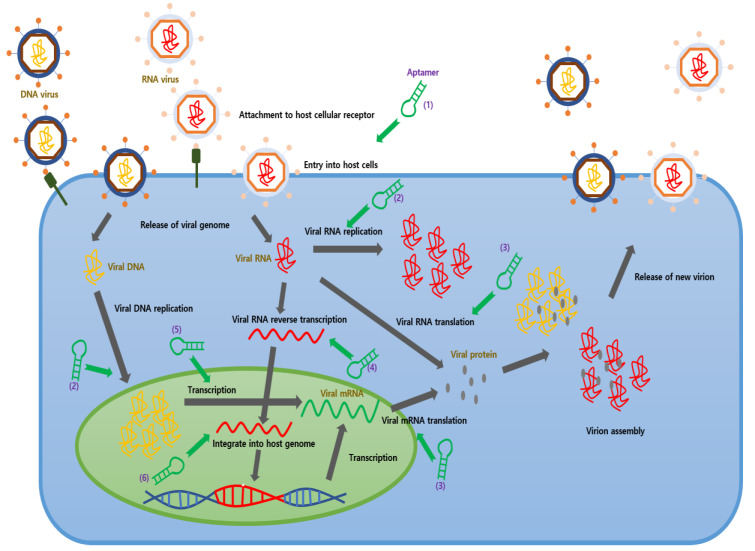
Scheme of viral life cycle and targets for aptamers within said cycle. The life cycle of a virus consists of attachment, entry, replication, assembly, and release. Aptamers can target factors involved in each step. (1) Aptamers can block viral entry into host cells by targeting host cell receptors or virus structural proteins. (2) Viral genome replication can be suppressed by targeting proteins such as viral polymerases and helicases. (3) Proteases, which are involved in the production of viral proteins, can be inhibited with aptamers thereby inhibiting viral replication. (4) The reverse transcription process can be prevented by targeting the reverse transcriptase involved in the RT of the viral RNA of retroviruses such as HIV. (5) Viral mRNA generation can be prevented by targeting factors involved in the transcription of the viral genome. (6) By targeting viral integrases, proviruses of viruses such as HIV can be prevented from being integrated into the genome of the host cell.

Compared with antibodies, the ability to bind to various targets with lower probabilities of immune response means that aptamers have major potential advantages for the treatment of viral diseases. Of note, the chemical synthesizability of aptamer confers one of the major advantages against antibody as therapeutics. In particular, with aptamers, it is possible to block various processes by specifically developing them to target various factors involved in the viral life cycle (Figure 2). In the case of HCV as an example, viral infection can be prevented by either targeting core proteins or envelope proteins, interrupting either the entry-stage or replication stage [43,44]. Alternatively, viral infection can be blocked through inhibition of viral replication by directly targeting the HCV RNA genome or by targeting NS5B, a viral RNA polymerase (Figure 2) [38,63,64]. In addition, the replication of the virus can be inhibited by targeting the MTase of JEV or DENV, which are other Flaviviridae viruses [77,80].

With consideration of recent pandemics including the pandemic caused by COVID-19, the development of fast and accurate diagnosis methods, appropriate treatments after diagnosis that are effective against a wide range of variants, and effective vaccines is urgently required. Due to advantages such as rapid development, high specificity, affinity, and stability, easy manufacturability, and convenient storage, aptamers could represent an excellent alternative to other current methods for the urgent purpose of controlling viral pandemics. However, as with all methods, there are obvious problems to be overcome. Aptamers can be selected by targeting various molecules, but there are cases where selection is prohibitively difficult. In addition, even if aptamers are appropriately selected, there are cases in which the results of aptamer methods differ between experimental testing and when used in clinical practice. Researchers are making ongoing efforts to overcome these shortcomings, such as modifying aptamers in various ways or developing improved SELEX methods [219]. Providing these shortcomings are overcome, aptamers will certainly be an attractive tool for dealing with viral diseases for the foreseeable future.

## Figures and Tables

**Table 1:** Comparison between aptamers and antibodies.

	Aptamer	Antibody
Material	Nucleic Acid (DNA, RNA)	Protein
Target	Wide range, including proteins	A limited target composed of amino acids
Size	~20 kDa	~150 kDa
Binding manner	3D structure recognition	Peptide sequence recognition
Immunogenicity	Minimal reported	Many reported
Development period	6–8 weeks	Months
Manufacturing	Chemical synthesis	Biological manufacturing
Storage	Room temperature	Cold temperature

**Table 2:** Summary data of anti-viral aptamers against virus types classified based on Baltimore classification, in this review.

Group	Virus	Target	Name of Aptamer	Type	Kd	Sequence	Applications	References
Group I	HPV	HPV-16 VLPs	Sc5-c3	RNA	0.05 pM	5′-GGG AAC AAA AGC UGC ACA GGU UAC CCC CGC UUG GGU CUC CCU AUA GUG AGU CGU AUU A-3′	Inhibition of HPV-16 infection	[9,11,17]
			HPV-07	DNA	400 ± 30 pM	n.d.	Specific detection of HPV-16	[10]
		HPV-16 E6/E7-HTECs	C5	RNA *	n.d.	5′-GGG AGG ACG AUG CGG AAG CAU CAA GGG UGA UCG UUU GAC CCU CCC CAG ACG ACU CGC CCG A-3′	Internalize aptamers into target cells	[15]
		E6	F2 and F4	RNA *	n.d.	5′-UGA UAA UAC GAC UCA CUA UAG GGA AUG GAU CCA CAU ACU ACG AAU AUU CAA CAU UCG AGG UGG AUG CUA CGA AUC AAC UUC ACU GCA GAC UUG ACG AAG CUU-3′ (F2)	Blocking E6–p53 interaction in cells	[16]
		E7	G5a3N	RNA	1.9 uM	5′-UAA UAC GAC UCA CUA UAG GGA GAC CCA AGC CGA UUU AUU UUG UGC AGC UUU UGU UCC CUU UAG UGA GGG UUA AUU-3′	Detection of HPV-16	[8]
			A2	RNA *	107 nM	5′-CCC UUC AUC AUU AAC CCG UCC ACG CGC-3′	Induce apoptosis in HPV infected cells	[12,13,14]
	HSV	gD protein	F2, D5 and G7a	RNA	n.d.	5′-GGG AGA GAG AGAG UCC AGU AAG CGC UCG UCA CACCCA CUA CGA GUG CCA UGC AUA UCU GCA ACA UCA CUU UAG GCC GAA UAA CGC AGA GGU AGA UGG-3′ (F2)	Inhibition of HSV-2 infection in cells	[19]
			aptamer-1 and -5	RNA *	109 nM	5′-AGU AAU ACG ACU CAC UAU AGG GCA CGA GAG AGG UCG UCC-3′ (aptamer-1)	Inhibition of HSV-1 entry in cells	[20]
			DApt	DNA	50 nM	5′-AGT AAT ACG ACT CA C TAT AGG GCA CGA GAG AGG TCG TCC-3′	Inhibition of HSV-1 entry in mice	[21]
Group IV	HCV	5′-UTR IRES	2-02 and 3-07	RNA	110 ± 10 nM	5′-GGG AGA AUU CCG ACC AGA AGG AGC GUU GCA GGG AAU GUA UGG CUA AUU CCC CUU UCC UCU CUC CUU CCU CUU CU-3′ (2-02)	Inhibition of HCV replication in vitro	[32,33,34,35,36]
		3′-UTR	cSL1, cSL2 and cSL3	RNA	n.d.	n.d.	Inhibition of HCV replication in vitro	[37]
			P-58 and P-78	RNA	0.47 ± 0.12 uM	5′-GGG AUA UUA UAG UAC AUA AAU GCC CCG CCU CAC AAA GGG AUG GGC UGU GGA GGU AGC GAA UUA AAG AGU AGU CGA A-3′ (P-58)	Inhibition of HCV replication in vitro	[38,39]
		Negative strand IRES	AP30	RNA	32 nM	5′-GUA CAC CAA AUC GCC CAC HCC CGU CUG GGA CUG GAU CAA C-3′	Inhibition of HCV replication in vitro	[40,41,42]
		Core	9-14 and 9-15	RNA *	100 nM	5′-GGG CCG UUC GAA CAC GAG CAU GUU GUC UAC GUU GUA GAA GCU GUU AUG GUA GGU ACU UCC ACG AGG UAU CAA CGG AGU UGG UGG ACA GUA CUC AGG UCA UCC UAG G-3′ (9-14)	Chip-based detection of HCV	[23]
			C4, 7, 42, 97, 103 and 104	DNA	n.d.	5′-GCA CGC CAG ACC AGC CGT CCT CTC TTC ATC CGA GCC TTC ACC GAG C-3′ (C4)	Inhibition of HCV production in cells	[44]
							GQ- based detection of HCV	[27]
			9-15	DNA	n.d.	5′-GAT CGA GGA TGG GAA CAC CCA GTA GGA GGA TGG GCA TGG CCG GAC CCA AAA TTA GCA GTA AAA AAA AAA AAA AA AAA-3′	LFA-based detection of HCV	[26]
			A12, A14 and A15	DNA	n.d.	5′-ACG CTC GGA TGC CAC TAC AGG CAC GCC AGA CCA GCC GTC CTC TCT TCA TCC GAG CCT TCA CCG AGC CTC ATG GAC GTG CTG GTG A-3′ (A12)	AFM-chip-based detection of HCV	[28,29]
		E2	ZE2	DNA	1.05 ± 1 nM	5′-GAA TGA GGA ATA ATC TAG CTC CTT CG CTG A-3′	Detection of HCV	[24]
			E2-A, B, C and D	DNA	0.8–4 nM	n.d.	Aptamer-based detection of HCV	[25]
		E1/E2	E1E2-6	DNA	n.d.	5′-CAC GTC TAT TAA GAT TGG GAC GTG-3′	Inhibition of HCV infection in cells	[43]
		NS2	NS2-2	DNA	n.d.	5′-CAG GTA CCA CCT TCA TGG GCG CGG AAG ACG ATG GTG TAC TA-3′	Blocking NS2–NS5A interaction in cells	[45]
			G6–16 and G6–19	RNA	120 ± 18 nM	5′-GGG AGA AUU CGA CCA GAA GCC UUG CUG UUG UUU CCC UGU UGU UUU GUC UCU CAA CUU UAU UGU GGU AAA GAU CAC UGG GUU GAU AAG GGC UAA CUC UAA UUU GAC UAC AUG GUC GGA CCA AUC AGU UCU UUG GGA GAU GCA UAU GUG CGU CUA CAU GGA UCC UCA-3′ (G6-16)	Inhibition of NS3 enzymatic activity in vitro	[46]
		NS3	SE RNA	RNA	990 pM	5′-GAA GCG UGC UGG GCC ACU AGU GUA UAC GGC UCG AA -3′	Inhibition of NS3 enzymatic activity in cells	[47]
			10G-1 and G9-I, II and III	RNA	8–12 nM	5′-GGG AAC UCG AUG AAG CGA AUU CUG UUG GCG AAC UGU ACG CAA GUA CAC UGG AUG ACA GCC UAC CUA UCG GAU CCA CG-3′ (10G-1)	Inhibition of NS3 protease activity in cells	[48,49,50,51]
			NEO-35-s41 and G925-s50	RNA	0.19 ± 0.16 nM	5′-CGU CCC CAA AAA AAA AGG AGA GAG GAA AGG UAG UC-3′ (NEO-35-s41)	Inhibition of NS3 enzymatic activity in cells	[54,55,56]
		NS5A	NS5A-4 and -5	DNA	n.d.	5′-GCT ATC TTA TGG AAA TTT CGT GTA GGG TTT GGT GTG GCG GGG CTA-3′ (NS5A-4))	Inhibition of HCV replication in cells	[57]
		NS5B	27v and 127v	RNA	132.3 ± 20 nM	5′-ACG TAC ACT AGT GGT CCG GGC GGG GCT CAT TGT CC-3′ (27v)	Inhibition of HCV replication in vitro and cells	[59,60]
			r10/43 and r10/47	DNA	1.3–23.5 nM	5′-GGG AGA CAA GAA TAA ACG CTC AAG GGC GTG GTG GGT GGG GTA ATA ATA ATG TGC GTT TGT TCG ACA GGA CCG TCA CAA CAG GC-3′ (r10/43)	Inhibition of HCV genotype 3a replication	[61]
			R-OH and R-F	RNA *	2.62 nM	5′-CCU UGA ACG AUU GGU AGU AGA AUA UCG UCA GUG AAC GGC AGU-3′ (R-F)	Inhibition of HCV replication in cells	[63,64]
	ZIKV	Capsid protein	n.d.	DNA	n.d.	n.d.	Detection of ZIKV using paper-based sensor	[70]
		NS1	Aptamers 2 and 10	DNA	45 pM	5′-CTA GGT TGC AGG GGA CTG CTC GGG ATT GCG GAT CAA CCT AG-3′ (Aptamer 2)	Detection of ZIKV using aptamer-based ELISA	[68]
				DNA	45 pM	5′-CTA GGT TGC AGG GGA CTG CTC GGG ATT GCG GAT CAA CCT AG-3′ (Aptamer 2)	Detection of ZIKV in serum and urine	[69]
	DENV	DENV	n.d.	DNA	n.d.	5′-CCC GCA CCG GGC AGG ACG TCC GGG GTC CTC GGG GGG CGG G-3′	Detection of DENV using nanoparticles	[74]
		Viral RNA	DEN-4 Linker	DNA	n.d.	5′-CGA GTT CAA CAT TCC TGT TTG CCC AAT CAT AGT TGA ACT CGT CTT G-3′	Detection of DENV using aptasensor	[73]
		5′-UTR	A03, B07 and C10	RNA	n.d.	5′-GGA GGU AGA GAG GGA GGG UUG AGG GGA AGG UUU ACC UCU UUA UUG-3′ (A03)	Potential for DENV diagnosis and therapy	[78]
		E protein	DENTA-1	DNA	99 ± 5 nM	5′-CGG CAT TCT CCT GCT ACG AGG CGC TGC GGT ACA CCC CGA CTC CAC GAG CCA CTG TCT ACG GAC ATC TG-3′	Inhibition of DENV infection	[75]
			S15	DNA	292 nM	5′-GCA CCG GGC AGG GAC GTC CGG GTC CTC GGG C-3′	Inhibition of 4 serotypes of DENV	[76]
		MTase	n.d.	RNA *	15.6 ± 1.03 nM	5′-GGU UGG GCA CAU AUA GAC UGU GUA AUU CGU AUA GUG UGC AUA ACC-3′	Inhibition of DENV MTase activity in vitro	[77]
	JEV	MTase	G2	RNA **	16 nM	5′-GAU GCG CAU GGA GAC GAC AGC AUC-3′	Inhibition of JEV MTase activity and in cells	[80]
	TBEV	E protein	n.d.	DNA	n.d.	n.d.	Inhibition of TBEV infection in cells	[82]
	NoV	VLPs	Aptamer 25	DNA	232 nM	5′-CAT CTG TGT GAA GAC TAT ATG GCG CTC ACA TAT TTC TTT C-3′	Detection of NoV using ELASA	[85]
			n.d.	DNA	n.d.	5′-GGG GGT TTT CAT CTG TGT GAA GAC TAT ATG GCG-3′	Detection of NoV using non-stop aptasensor	[90]
			AG3	DNA	290 nM	5′-GCT AGC GAA TTC CGT ACG AAG GGC GAA TTC CAC ATT GGG CTG CAG CCC GGG GGA TCC-3′	Detection of Nov using DNA aptasensor	[88]
		Capsid protein	M1 and M6-2	DNA	n.d.	5′-TGT TTA TGG GGA TAA ACG TAT CTA ATT CGT GTA CTA ATC A-3′ (M1)	Detection of NoV using ELASA	[86]
			APTL-1	DNA	148.13 ± 6.53 nM	5′-CGA TCA AAC GTT CAA GCG GGG CCC GGA GGCGTG ACT TGG ACA GGC AGG CGT TAC GAT GCA TCC CGC AAA TGA CGC ATG A-3′	Detection of NoV in clinical samples	[87]
			n.d.	DNA	n.d.	5′-AGT ATA CCG TAT TAC CTG CAG CCA TGT TTT GTA GGT GTA ATA GGT CAT GTT AGG GTT TCT GCG ATA TCT CGG AGA TCT TGC-3′	Detection of NoV using microfluidic platform	[89]
	CoV	SARS-CoV nsP10	ES15	RNA	n.d.	5′-GAU AAU ACG ACU CAC UAU AGG GUU CAC UGC AGA CUU GAC GAA GCU UGC AGA AAA GGG GGA AGA AGA GGG UGA UUC AGG CGA GAG AAU GGA UCC ACA UCU ACG AAU UC-3′	Inhibition of helicase activity in vitro	[94]
			NG1	DNA	20.8 nM	5′-CCG TAA TAC GAC TCA CTA TAG GGG AGC TCG GTA CCG AAT TCG TGT GAG GGT GAG ATG TGT GTG TAT TTG TCA AGC TTT GCA GAG AGG ATC CTT-3′	Inhibition of helicase activity in vitro	[95]
		SARS-CoV NP	Aptamer 1 and 2	RNA *	1.65 ± 0.41 nM	5′-GGG AGA GCG GAA GCG UGC UGG GCG UGU CGU UCG CUG UCU UGC UAC GUU ACG UUA CAC GGU UGG CAU AAC CCA GAG GUC GAU GG-3′ (Aptamer 1)	RNA aptamer-based detection of SARS-CoV	[96]
			Aptamer1	DNA	4.93 ± 0.30 nM	5′-GCA ATG CTA CGG TAC TTC CGG ATC CGG AAA CTG GCT AAT TGG TGA GGC TGG GGC GGT CGT GCA GCA AAA GTG CAC GCT ACT TTG CTA A-3′	Detection of SARS-CoV	[97]
			n.d.	RNA	n.d.	5′-GGG AGA GCG GAA GCG UGC UGG CCC UGU CGU UCG CUG UCU UGC UAC GUU ACG UUA CAC GGU UGG CAU AAC CCA CAG GUC GAU GG-3′	Detection of SARS-CoV using quantum-dots	[98]
		SARS-CoV2 spike	CoV2-RBD-4C	DNA	19.9 nM	5′-ATC CAG AGT GAC GCA GCA TTT CAT CGG GTC CAA AAG GGG CTG CTC GGG ATT GCG GAT ATC GAC ACG T-3′	Provide new strategy to SARS-CoV2	[99]
		SARS-CoV2 NP	A48	DNA	0.49 nM	5′-GCT GGA TGT CGC TTA CGA CAA TAT TCC TTA GGG GCA CCG CTA CAT TGA CAC ATC CAG C-3′	Provide new strategy to SARS-CoV2	[100]
Group V	Influenza Virus	H1N1 virus	Apt-DNA	DNA	n.d.	5′-ACA CAA ATC CTA TTG ACC GCT GTG TGA CGC AAC ACT CAA T-3′	Detection of virus using quantum dots (QDs)	[108]
			n.d.	DNA	n.d.	5′-GGC AGG AAG ACA AAC AGC CAG CGT GAC AGC GAC GCG TAG GGA CCG GCA TCC GCG GGT GGT CTG TGG TGC TGT-3	Detection of virus using microfluidic system	[3,109]
			A-20	DNA	6 nM	5′-GGA CCA GTT GTC TTT CGG TCT CTA CCC CAG CCC GT-3′	Detection of virus using EIS	[111]
			AP-I	DNA	n.d.	5′-GCA ATG GTA CGG TAC TTC CGG GTG GGT GGG AGG GGG TGG AGG TTG GGG GTT GGA CGC AGA GTG CCA AAA GTGC ACG CTA CTT TGC TAA-3′	Distinguish virus subtype H1 form H5	[110]
		H1N1 HA	V46	DNA	19.2 nM	5′-TAC TGC ACA CGA CAC CGA CTG TCA CCA TCA CCT CGG CGC A-3′	Detection of virus by electrochemical sensor	[112]
			n.d.	DNA	n.d.	5′-GGG TTT GGG TTG GGT TGG GTT TTT GGG TTT GGG TTG GGT TGG GAA AAA-3′	Detection of virus using SERS aptasensor	[113]
			Aptamer 1 and 2	DNA	78 ± 1 nM	5′-GGG AGC TCA GAA TAA ACG CTC AAG GCA CGG CAT GTG TGG TAT GTG GTG CCT GTA CTC GTT CGA CAT GAG GCC CGG ATC-3′ (Aptamer 1)	Blocking HA–Glycan interaction in cells	[137]
			D-12 and D-26	RNA *	190 pM	5′-GGA GCU CAG CCU UCA CUG C CA AAA AGU UAG GCC AGC AAA UUG CGA GCU GAU CCG GUG ACU GGC UAC AGG AGG CCU UGU CCA CGG CCG UAU U GG CAC CAC CGU CGG AUC C-3′ (D-12)	Inhibit agglutination of virus	[129]
		H3N2 HA	A22	DNA	46.23 ± 5.46 nM	5′-GCT GCA ATA CTC ATG GAC AGC CTC CTG GGG TCA GGC TCA GAC ATT GAT AAA GCG ACA TCG GTC TGG AGT ACG ACC CTG AA-3′	Inhibition of viral infection in mice	[136]
							Detection of virus using QDs	[107]
							Detection of virus using colorimetric platform	[115]
			P30-10-16	RNA	n.d.	5′-GGG AGA AUU CCG ACC AGA AGG GUU AGC AGU CGG CAU GCG GGU CAC GAC AGA CCU UUC CUC UCU CCU UCC UCU UCU-3′	Inhibition of HA-mediated entry of virus	[128]
							Detection of virus using aptasensor	[114]
							Detection of virus using DRELFA	[116]
			RHA0385	DNA	n.d.	5′–TTG GGG TTA TTT TGG GAG GGC GGG GGT T–3′	Detection of virus using SERS aptasensor	[117]
		H5N1 HA	Aptamer 1, 2 and 3	DNA	4.65 nM	5′-GTG TGC ATG GAT AGC ACG TAA CGG TGT AGT AGA TAC GTG CGG GTA TGT TG-3′ (Aptamer 1)	Detection of virus using SPR aptasensor	[118]
							Detection of virus using QCM aptasensor	[119]
							Detection of virus using microfluidic chip	[120]
							Detection of virus using gold nanoparticles	[122]
			RHA0006	DNA	n.d.	3’-TTG GGG TTA TTT GGG AGG GCG GGG GTT-5’	Detection of virus using fluorescent sensor	[121]
			IF10 and IF22	DNA	n.d.	5′-CGT ACG GTC GAC GCT AGC TAA CGG TGT GGC CCG GGG GTA CAG CGC ACT CAC GTG GAG CTC GGA TCC-3′ (IF10)	Detection of virus using SPR aptasensor	[123]
			n.d.	DNA	n.d.	5′-GTG TGC ATG GAT AGC ACG TAA CGG TGT AGT AGA TAC GTG CGG GTA GGA AGA AAG GGA AAT AGT TGT CCT GTT GTT GCC ATG TGT ATG TGG G-3′.	Detection of virus using FET	[124]
			HAS15-5	RNA	n.d.	5′-GGG TTC ACT GCA GAC TTG ACG AAG CTT ACA AAC AAG AGC AAA AAG GGA GUU GAC GUA GAC UGU GCG GAA TGG ATC CAC ATC TAC GAA TTC-3′	Blocking HA activity	[132]
			BV02	DNA	n.d.	5′-AAT TAA CCC TCA CTA AAG GGC TGA GTC TCA AAA CCG CAA TAC ACT GGT TGT ATG GTC GAA TAA GTT AA-3′	Inhibition of viral replication in animal model	[133]
			HA12-16	RNA	n.d.	5′-GGG TTC ACT GCA GAC TTG ACG AAG CTT GCU UGA CGG AGA UCA AGG GCG AGU CUC AUA CCA AGU UGA UGG GGA ATG GAT CCA CAT CTA CGA ATT C-3′	Inhibition of viral replication in cells	[134]
			8-3S	RNA*	170 pM	5′-GGG CAA CCG CUG GAA CUU GAA GUC GGU AAU GCG AGC GGA AAG CCC-3′	Blocking HA–Glycan interaction	[135]
		H5N1 pA	PAN-1 ~ PAN-6	DNA	130–2000 nM	5′-CCG TAA TAC GAC TCA CTA TAG GGG AGC TCG GTA CCG AAT TCC TTG GAC CAT TAA AAC ACG TGT CTG CAT CCA AGC TTT GCA GAG AGG ATC CTT-3′ (PAN-1)	Inhibition of endonuclease activity	[140]
		H9N2 HA	C7	DNA	n.d.	5′-GGT AGT TAT AGT ATA TGG AAG GGG GTG TCG TAT GG-3′	Inhibition of viral infection in cells	[131]
			A9 and B4	DNA	46.23 ± 5.46 nM	5′-GCT GCA ATA CTC ATG GAC AGC CTC CTG GGG TCA GGC TCA GAC ATT GAT AAA GCG ACA TCG GTC TGG AGT ACG ACC CTG AA-3′ (A9)	Inhibition of viral infection in cells	[136]
		Influenza A NS1	n.d.	DNA	18.91 ± 3.95 nM	5′-GCA ATG GTA CGG TAC TTC CCG CGG TCC GGG GTG GGT GGG TGG TGG GGG GTG CGG GGG GGC GGC CGC AAA AGT GCA CGC TAC TTT GCT AA-3′	Inhibition of interferon antagonism	[138]
		Influenza B HA	A-20	RNA	45 nM	5′-GGG UGG ACG CGG UAC GAG CAA UUU GUA CCG GAU GGA UGU UCG CC-3′	Inhibition of viral entry	[127]
	RVFV	NP	n.d.	RNA	n.d.	5′-GGU AGC CAU AUU AGC GCA UAA CCA UCA CAA CCG UGG GCU CAU UGG UGG CCA CUG CCA U-3′	Inhibition of viral replication	[147]
	SFTSV	NP	SFTS-apt 1, 2 and 3	DNA	1–4 nM	5′-ATC CAG AGT GAC GCA GCA CGA CCA CAG ATT GGA GAC TGA TAG TGC ACG AGC AAG GAC ATG GAC ACG GTG GCT TAG T-3′ (SFTS-apt 1)	Detection of virus using aptasensor	[151]
	EBOV	VP35	1G8-14 and 2F11-14	RNA	3.7 ± 0.2 nM	5′-GGG AGA CAA GAA UAA ACG CUC AAG GCA UUU CUG CUA GUC UGG UUG UAA GAU AUU CAA CAC GUG AGU UUC GAC AGG AGG CUC ACA ACA GGC-3′ (1G8-14)	Inhibition of VP35–NP interaction	[153]
		VP24	VPKS-2 and VPKS-5	DNA	0.5–20 nM	n.d.	Inhibition of VP24–KPNA1 interaction	[154]
		GP	39SGP1A	RNA *	30 nM	5′-GGG CGC UCA AUU UUU UAU UGC AUU UUU CUU UGA GCG CCC-3′	Detection of viral infection	[155]
		GP and NP	n.d.	DNA	4.1–76.1 nM	5′-GGG CGC UCA AUU UUU UAU UGA GCG CCC-3′	Detection of viral infection	[156]
Group VI	HIV	Genome	AL4 and AS8	RNA	n.d.	5′-GGG AGU CGA CCG ACC AGA AAG CUA GGG AAC AGG GGA GGA GCA GGC AGU AGG UGC GAU GGU AUG UGC GUC UAC AUC UAG ACU CAU-3′ (AL4)	Inhibition of viral transcription	[167]
			RNApt16	RNA	82 ± 13 nM	5′-CCC CGG CAA GGA GGG G-3′	Inhibition of viral production in cells	[168]
		RT	RT 26	DNA	n.d.	n.d.	Detection of virus	[161]
			n.d.	DNA	25 pM	5′-GGG AGA UUC CGU UUU CAG UCG GGA AAA ACU GAA-3′	Inhibition of viral production in cells	[170]
			Class1 and 2	RNA	n.d.	5′-CAC AAG AUC CGA GGC AGA ACG GGA AAA UCU GCG AAG UAA-3′ (Class 1)	Inhibition of DNA polymerase activity	[171,172]
			70.8 and 70.15	RNA	27–63 nM	n.d.	Inhibition of viral replication in cells	[173,174]
			RT1t49	DNA	4nM	5′-ATC CCT GAT TAG CGA TAC TCA GAA GGA TAA ACT GTC CAG AAT TTG GA-3′	Inhibition of polymerase and RNaseH activity	[175,176]
							Inhibition of viral infection in cells	[180]
			n.d.	DNA	5nM	5′-GCA TGA ATT CCC CGA AGA CGC AAA CTG AAG AGG CAC CGA AGG GGG GG-3′.	Inhibition of viral replication	[177]
			RT6	DNA	n.d.	5′-CAG GCG TTA GGG AAG GGC GTC GAA AGC AGG GTG GG-3′	Inhibition of RT activity	[178]
			70.15	RNA	n.d.	5′-ACC CAG GAG AUA AAG GGG AAA ACA CUG GAA AAC-3′	Inhibition of viral infection in cells	[179]
			F1Pk and F2Pk	RNA	n.d.	5′-CCU AGG ACG AAA GCG AUA AUC GGG CCU GGA GGA UCA AAU UAA UGC U-3′ (F1Pk)	Inhibition of viral replication	[181]
			148.1-38m	RNA*	n.d.	5′-GGG CGU UGC CUA CUC UCA AUC UGA GGU UCA AGG GCA CG-3′	Inhibition of viral infection	[183]
			ODN 93 and ODN 112	DNA	n.d.	5′-GGG GGT GGG AGG AGG GTA GGC CTT AGG TTT GTG A-3′ (ODN 93)	Inhibition of RNaseH activity	[184,185]
			ODN	DNA	n.d.	5′-GGG GGG GCC AGG CCA TGG CGT GAC TTG CTG GC-3′	Inhibition of integrase activity	[187]
		IN	T30177	DNA	n.d.	5′-GTG GTG GGT GGG TGG GT-3′	Inhibition of integrase activity	[188]
			93del	DNA	n.d.	5′-GGG GTG GGA GGA GGG T-3′	Inhibition of viral replication ex vivo	[190]
			T30175	DNA	n.d.	5′-GHG GTG GGT GGG TGG GT-3′	Inhibition of integrase activity	[191]
		PR	PR10.1	RNA	115 ± 22 nM	5′-CUU CAU UGU AAC UUC UCA UAA UUU CCC GAG GCU UUU ACU UUC GGG GUC CU-3′	Inhibition of viral production in cells	[193]
		NC	N70-13	RNA	0.6 nM	5′-GAC UGG GUA CGU UUC CGG UAG CCG GUA GGA-3′	Inhibition of viral packaging	[195,196]
		Gp120	B40	RNA *	20.9 nM	5′-GGG AGA CAA GAC UAG ACG CUC AAU GUG GGC CAC GCC CGA UUU UAC GCU UUU ACC CGC ACG CG-3′	Inhibition of viral infection in cells	[198,199,200,201,202]
		Gag	DP6-12	RNA	130 ± 9.3 nM	n.d.	Inhibition of viral infection in cells	[204]
		Rev	n.d.	n.d.	n.d.	n.d.	Inhibition of viral infection in cells	[207]
			RBA-14	RNA	5.9 nM	5′-GGC UGG ACU CGU ACU UCG GUA CUG GAG AAA CAG CC−3′	Inhibition of viral infection in cells	[205]
		Tat	n.d.	RNA	n.d.	5′-ACG AAG CUU GAU CCC GUU UGC CGG UCG AUC GCU UCG A-3′	Detection of virus using aptasensor	[162,163]
			n.d.	RNA	n.d.	5′-UCG GUC GAU CGC UUC AUA A-3′	Detection of virus using FET-based sensor	[164]
			AntiTat5	RNA	n.d.	5′-ACG AAG CUU GAU CCC GUU UGC CGG UCG AUC GCU UCG AAA AAA AAA AAA CGA AGC UUG AUC CCG UUU GCC GGU CGA UCG CUU CG-3′	Detection of virus using SE-based sensor	[166]
		CCR5	G3	RNA	110 nM	5′-GCC UUC GUU UGU UUC GUC CA-3′	Inhibition of viral infection in cells	[208]
		NCL	AS1411	DNA	34.2 nM	5′-GGT GGT GGT GGT TGT GGT GGT GGT GG-3′	Inhibition of viral entry to cells	[209]
		CycT1	Apt1 and Apt4	RNA	1–2 nM	5′-UCC CCC UAU GCG AAA AGC GAA UCA CUU CCA GCC UAC CCU-3′ (Apt1)	Inhibition of viral transcription in cells	[211]
Group VII	HBV	HBsAg	anti-HBsAg RNA aptamer	RNA	n.d.	5′-GUA UGU GGG CUG AAC UCA AUC AGG UCC CAA UCC CCA ACA UAC ACA UGA CCC GUC GUU UAC GAU CAU UAU AGA CGG CCA UGA UUG ACA CGC AAU CAA CCC CCU AUA GUG AGU CGU AUU A-3′	Detection of virus using FRET	[212]
			HO1, HO2 and HO3	DNA	n.d.	5′-GGG AAT TCG AGC TCG GTA CCC ACA GCG AAC AGC GGC GGA CAT AAT AGT GCT TAC TAC GAC CTG CAG GCA TGC AAG CTT GG-3′ (HO1)	Detection of virus using MNPs	[213]
			H01	DNA	n.d.	5′-ACC CAC AGC GAA CAG CGG CGG ACA TAA TAG TGC TTA CTA CGA CGC-3′	Remove HBsAg in cell with no cytotoxicity	[218]
		HBeAg	Aptamer 2-19	DNA	n.d.	5′ -GGG CGA AGA CCG GGA CGG GAG GAT TCT GTA GAT TGG TTT T-3′	Detection of virus	[214]
		Polymerase	Class I and Class II	RNA	n.d.	5′-UGU UCA UGU CCU ACU GUU CCG AAC AAA AAU AAG AAG AAA AAU AAU AUU UGG GGC AUG GAC A-3′ (Class 1)	Inhibition of viral replication in cells	[215]
		Core	Apt No 28	DNA	n.d.	5’-ACG CTC GGA TGC CAC TAC AGC TTC CCC TAA TCT GGC GCT CTC ATC TAA TTT CCC TTC CTG CTC ATG GAC GTG CTG GTG AC-3’	Inhibition of viral infection in cells	[216]
		capsid	AO-01	DNA	180 ± 82 nM	5′-GCG GGT CGA CGT TTG CAC ACG CGA GCC GCC ATG TCT GGG CCA CAT CCA TGG GGC GG-3′	Inhibition of viral production in cells	[217]

n.d. = not determined, * = 2′-fluoro-pyrimidine modified aptamer, ** = 2′-O-methyl-pyrimidine modified aptamer.
